# Hepatic Phenotype in NBAS‐Associated Disease: Clinical Course, Prognostic Factors and Outcome in 230 Patients

**DOI:** 10.1111/liv.70146

**Published:** 2025-05-28

**Authors:** Bianca Peters, Lea Dewi Schlieben, Heiko Brennenstuhl, Cigdem Arikan, Sarah M. Bedoyan, Fatma Derya Bulut, Ellen Crushell, Carlo Dionisi‐Vici, Ada Drab, Alexander Fichtner, Aixa Gonzalez Garcia, Deanna Fry, Sven F. Garbade, Nicole Hammann, Nedim Hadzic, Robert Hegarty, Marianne Hørby Jørgensen, Martin Laaß, Elke Lainka, Lina Leghlam, Eberhard Lurz, Halise Neslihan Önenli Mungan, Andrea Pietrobattista, Begona Polo, Piotr Socha, James E. Squires, Tian Sun, Georg F. Vogel, Holger Prokisch, Stefan Kölker, Georg F. Hoffmann, Christian Staufner, Dominic Lenz

**Affiliations:** ^1^ Division of Pediatric Neurology and Metabolic Medicine, Department of Pediatrics I, Medical Faculty of Heidelberg Heidelberg University Heidelberg Germany; ^2^ European Reference Network on Hepatological Diseases (ERN RARE‐LIVER) Hamburg Germany; ^3^ European Reference Network on Hereditary Metabolic Disorders (MetabERN) Udine Italy; ^4^ School of Medicine, Institute of Human Genetics, Klinikum Rechts der Isar Technical University of Munich Munich Germany; ^5^ Institute of Neurogenomics, Computational Health Centre Helmholtz Munich Munich Germany; ^6^ Institute of Human Genetics Heidelberg University and University Hospital Heidelberg Heidelberg Germany; ^7^ Department of Pediatric Gastroenterology and Organ Transplant Koc University School of Medicine Istanbul Turkey; ^8^ Department of Pediatric Gastroenterology and Hepatology UPMC Children's Hospital of Pittsburgh Pittsburgh Pennsylvania USA; ^9^ Division of Pediatric Metabolism, Department of Pediatrics Cukurova University Faculty of Medicine Adana Turkey; ^10^ National Centre for Inherited Metabolic Disorders Childrens Health Ireland Dublin Ireland; ^11^ Division of Metabolic Diseases and Hepatology Bambino Gesù Children's Hospital IRCCS Rome Italy; ^12^ Department of Gastroenterology, Hepatology, Feeding Disorders and Pediatrics The Children's Memorial Health Institute (CMHI) Warsaw Poland; ^13^ University of Arkansas for Medical Sciences (UAMS) and Arkansas Children's Hospital Little Rock Arkansas USA; ^14^ Pediatric Liver, GI and Nutrition Centre King's College Hospital London UK; ^15^ Department of Pediatric and Adolescent Medicine Rigshospitalet Copenhagen Denmark; ^16^ Department of Pediatrics, Faculty of Medicine and University Hospital Carl Gustav Carus Technische Universität Dresden Dresden Germany; ^17^ Pediatrics II, Department for Pediatric Nephrology, Gastroenterology, Endocrinology and Transplant Medicine, University Hospital Essen University of Duisburg‐Essen Essen Germany; ^18^ Department of Pediatrics, Dr. von Hauner Children's Hospital, University Hospital LMU Munich Munich Germany; ^19^ Pediatric Gastrohepathology Unit Hospital Universitario y Politécnico La Fe Valencia Spain; ^20^ Department of Paediatrics I Medical University of Innsbruck Innsbruck Austria; ^21^ Institute of Cell Biology, Biocenter Medical University of Innsbruck Innsbruck Austria

**Keywords:** disorders of intracellular trafficking, genetic liver disease, infantile liver failure syndrome type 2, NBAS, recurrent acute liver failure

## Abstract

**Background and Aims:**

Since described in 2015, NBAS‐associated disease has emerged as an important cause of acute liver failure (ALF) in children. We analysed the variable expression, genotype–phenotype association, outcome and prognostic factors of the hepatic involvement.

**Methods:**

Individuals with biallelic pathogenic *NBAS* variants were recruited within an international observational study, including new and previously published patients.

**Results:**

We studied 230 individuals, including 13 previously unreported patients. The liver was the most frequently affected organ (63.4%), with 41.3% experiencing at least one ALF. The median age at onset was 0.9 years, the median age at last ALF 5 years, the latest ALF occurred at 24 years. Liver crises were triggered by febrile infections and presented with highly increased hepatic transaminases. Liver involvement varied significantly between the subgroups: 91.7% of patients with infantile liver failure syndrome type 2 and 88.9% of patients from the combined subgroup (variants affecting β‐propeller domain) presented with ALF, whereas SOPH (stature, optic atrophy, Pelger–Huët anomaly) patients mostly had either no liver involvement (66.4%) or persistently elevated transaminases without ALF (28%). The rate of native liver survival was 83.9%; 16 individuals underwent liver transplantation and 24 died.

**Conclusion:**

Liver abnormalities are common and the leading cause of death in NBAS‐associated disease. There is a clear genotype–phenotype association regarding the hepatic involvement. Liver crises occur primarily during infancy; however, early medical attention in case of febrile infections is necessary at all ages. Liver transplantation prevents ALF, but its risks must be weighed against the frequency and severity of liver crises decreasing with age.

**Abbreviations**
ALF: acute liver failure
ALT: alanine aminotransferase
AST: aspartate aminotransferase
ELT: elevated liver transaminases
HE: hepatic encephalopathy
IgG: immunoglobulin G
ILFS2: infantile liver failure syndrome type 2
INR: international normalised ratio
NAC: N‐acetylcysteine
NBAS: neuroblastoma amplified sequence
NK cell: natural killer cell
NLS: native liver survival
NNLS: non‐native liver survival
PELD: paediatric end‐stage liver disease
SOPH: short stature, optic atrophy and Pelger–Huët anomaly

**Summary**

- NBAS‐associated disease is a multisystemic disorder, with liver abnormalities being the most prevalent symptom and the primary cause of mortality.
- Liver crises mainly occur within the first decade of life; however, awareness and early medical attention in case of febrile infections are necessary across all ages.
- Liver transplantation effectively prevents liver failure but carries significant risks of morbidity and mortality.

## 1 Introduction

Liver disease encompasses a wide range of conditions and is an important cause of morbidity and mortality in children worldwide [1]. While environmental factors such as viral hepatitis and intoxication are well‐established contributors to acute liver disease, genetic disorders also play a major role in liver disease, especially in children. New diagnostic methods, in particular next‐generation sequencing, have significantly improved the diagnostic yield for liver disease [2, 3] and enabled the identification of previously unknown genetic causes. A novel important group is disorders of intracellular trafficking, with neuroblastoma amplified sequence (NBAS)‐associated disease being the most prominent member [4]. It has been identified as a relatively frequent cause of liver dysfunction and, in particular, recurrent acute liver failure (ALF) [5, 6, 7]. A specific homozygous variant in *NBAS* was first described in 2010 in the Yakut population as the cause of short stature, skeletal abnormalities, optic atrophy and Pelger–Huët anomaly (SOPH syndrome; MIM: 614800) [8]. In 2015, biallelic variants in *NBAS* were associated with recurrent ALF, termed infantile liver failure syndrome type 2 (ILFS2; MIM: 616483) [5]. Since then, more than 200 affected individuals have been reported, confirming that NBAS‐associated disease is a multisystem disorder [7, 9, 10]. Besides liver, skeleton and growth, the immune, nervous and endocrine systems are frequently affected. In 2020, three phenotypic subgroups were defined based on the affected region of the NBAS protein [7]. Patients with variants affecting the Sec39 domain show a predominantly hepatic phenotype with recurrent ALF (ILFS2 subgroup). Patients with alterations in the C‐terminal protein segment present with a multisystemic phenotype with skeletal, facial, immunological, ophthalmological, neurological, and mild hepatic involvement (SOPH syndrome subgroup). Variants affecting the β‐propeller domain cause a severe combined phenotype with ALF as well as multisystem involvement (combined subgroup).

In this study, we systematically and quantitatively analyse the multifaceted presentation, clinical course, outcome, and predictive factors of liver involvement in NBAS‐associated disease.

## 2 Patients and Methods

Individuals were recruited within an international, observational study. Inclusion criteria were biallelic variants in *NBAS* (NM_015909.3) classified as pathogenic or likely pathogenic according to American College of Medical Genetics (ACMG/AMP) criteria [11]. Patients were excluded from the quantitative analysis if they fulfilled one of the following criteria: [1] presence of severe comorbidities unrelated to *NBAS* variants, [2] presymptomatic genetic diagnosis, and [3] lack of data about the hepatic phenotype.

Patient data was collected via a case report form or retrieved from publications. For the identification of previously published patients, a comprehensive literature search was performed with PubMed and Google Scholar using the terms ‘NBAS AND/OR SOPH’ and ‘NBAS AND/OR ILFS2’ on June 14, 2024. Data from the case report forms and literature search are stored in a disease‐specific database located at the University Hospital Heidelberg. The database was established in 2018 and is regularly updated. Some data have already been included in other publications [7, 12, 13]. Identification by ID number (‘NBAS‐ID’) has been continued from previous publications. Patients were assigned to three genetic subgroups based on the type and localisation of the *NBAS* variants as proposed previously [7]. ALF was defined according to the inclusion criteria of the PALF study group [14].

All procedures followed were in accordance with the ethical standards of the responsible committee on human experimentation (institutional and national) and with the Helsinki Declaration of 1975, as revised in 2013. Informed consent was obtained from all patients or their parents or caregivers in the case of minor patients, except for cases where patient data were retrieved from publications. The study was approved by the ethical committee of the Medical Faculty Heidelberg (study number: S‐035_2014).

Statistical analysis was performed using GraphPad Prism version 10 and ‘R’ version 4.0.4 (R: A language and environment for statistical computing. R foundation for Statistical Computing, Vienna, Austria). Continuous variables were described using median and range. Categorical variables were described using frequencies and percentages. The number n indicates the number of individuals for whom the respective information was available. Due to the variable phenotype between family members, all single individuals were included in the analysis and not reduced to family cases. Chi‐square statistics were used to test for differences in percentages of categorical variables. Wilcoxon rank sum/Mann Whitney test and Kruskal–Wallis test were used to compare ordinal scaled data between different groups. Dunn's Bonferroni tests were used for multiple comparisons. Kaplan–Meier estimates and log‐rank test were applied to right‐censored data. *p*‐values less than 0.05 were considered statistically significant.

## 3 Results

### 3.1 Study Population

The overall cohort includes a total of 244 patients with biallelic (likely) pathogenic *NBAS* variants. Literature search identified 59 publications and 5 conference abstracts with a total of 231 patients, published between February 2008 and May 2024 [3, 5, 6, 8, 9, 10, 12, 13, 15, 16, 17, 18, 19, 20, 21, 22, 23, 24, 25, 26, 27, 28, 29, 30, 31, 32, 33, 34, 35, 36, 37, 38, 39, 40, 41, 42, 43, 44, 45, 46, 47, 48, 49, 50, 51, 52, 53, 54, 55, 56, 57, 58, 59, 60, 61, 62, 63, 64, 65, 66, 67, 68, 69, 70]. Data on 13 patients were not previously published. For 70 patients, who have already been reported in previous publications, unpublished data on the hepatic phenotype was included. After evaluation for exclusion criteria, 230 patients were included in the quantitative analysis.

Five patients were excluded due to additional comorbidities unrelated to the *NBAS* variants (see Supporting Information, methods for more details).

One patient (NBAS 71; last visit at the age of 5 years) was diagnosed by family screening and, under early antipyretic therapy, showed no NBAS‐associated symptoms other than the Pelger‐Huët anomaly [34]. For six patients from the literature search, no information on the hepatic phenotype was available, and the patients were therefore excluded (NBAS 125, [68]; NBAS 143 [71], NBAS 126–128 [3], NBAS 162; 163 [72] and NBAS 49 [69]).

Patients originate from all continents and 30 different countries, with Russia (95 patients) and China (29 patients) being the most represented countries. Yet, 91 of the Russian patients live isolated in Yakutia in north‐eastern Siberia. The high prevalence of a specific *NBAS* variant (c.5741G>A; p.(Arg1914His)) in the Yakut population has been attributed to a founder effect [73].

The study cohort includes 130 female and 100 male patients from 205 families. Age at last visit ranges from 4 months to 53 years, with a median age of 9 years. Taken together, a total of 2681 patient years have been studied. Dunn's Multiple Comparison Test showed that age at last visit differed significantly between SOPH subgroup (median 14 years, range 0.4–53 years) and ILFS2 subgroup (median 5.3 years, range 0.33–45 years). The median age at last visit in the combined subgroup was 9 years (range 1.5–23 years) (Table 1).

**TABLE 1:** Overview of gender and hepatic phenotypes. Subgroup results that differ from the expected frequencies in the Chi‐square test (Pearson residuals > |1.96|) are marked with *. Ages were compared using the Kruska–Wallis test. Percentages are always calculated in relation to data availability (last row).

	All patients	Genetic subgroups				*p*	Data availability
		Combined	ILFS2	SOPH	No subgroup		
Total number	230	18 (7.8%)	48 (20.9%)	125 (54.3%)	39 (17%)		230
Female	130 (56.5%)	10 (55.6%)	26 (54.1%)	66 (52.8%)	28 (71.8%)		230
Age at last visit in years; median (range)	9 (0.33–53)	9 (1.5–23)	5.3 (0.33–45)	14 (0.4–53)	4 (0.33–28)	**< 0.0001**.	228
Abnormality of the liver	146 (63.5%)	18 (100%)*	48 (100%)*	42 (33.6%)*	38 (97.4%)*	**< 0.0001**	230
Acute liver failure	95 (41.3%)	16 (88.9%)*	44 (91.7%)*	7 (5.6%)*	27 (69.2%)*	**< 0.0001**	224
Non‐native liver survival	34 (14.8%)	7 (38.9%)*	16 (33.3%)*	4 (3.2%)*	7 (17.9%)	**< 0.0001**	230
Age at first ELT crisis/ALF episode in years; median (range)	0.9 (0–11)	1 (0–3)	1.1 (0.17–3.25)	0.5 (0–11)	0.9 (0–7)	0.5839	107
Number of ALF episodes	0 (0–12)	2 (0–8)	2 (0–12)	0 (0–8)	2 (0–6)	**< 0.0001**	108
Age at last ALF episode in years; median (range) only including patients > 5 years without ALF	5 (2–24)	4.5 (2–14)	4.8 (2.16–24)	n.a.	6.5 (2.5–9)	0.73	27
Age at most severe ELT/ALF in years; median (range)	2.2 (0–12)	2.8 (0–12)	2.3 (0.75–7)	1.9 (0.42–5)	1.4 (0.02–6.67)	0.1609	92
Normalisation of ALAT/ASAT between episodes	77 (72.6%)	14 (82.4%)	34 (91.9%)*	11 (42.3%)*	18 (69.2%)	**0.0002**	106
Hepatic encephalopathy	66 (36.1%)	11 (68.8%)*	35 (89.7%)*	3 (2.8%)*	17 (81%)*	**< 0.0001**	183
AST (max.); median (range)	8920 (59–13 790)	7686 (3234–27 630)	12 213 (225–13 790)	1367 (59–22 546)	8103 (65–24 780)	**< 0.0001**	99
ALT (max.); median (range)	7830 (98–22 655)	9500 (4531–14 500)	8058 (178–22 655)	790 (98–12 392)	8270 (208–21 483)	**< 0.0001**	105
INR (max.); median (range)	5.2 (1–30)	2.35 (1–9.88)	8.03 (2.02–30)	1.91 (1.08–5.22)	3.9 (1.2–16)	**< 0.0001**	76

*Note*: Bold was used for the *p* values when statistical significance was reached.

Abbreviations: ILFS2, infantile liver failure syndrome type 2; SOPH, short stature, optic atrophy and Pelger–Huët anomaly.

### 3.2 Genetics and Subgroups

A total of 158 different *NBAS* variants were identified; variants are distributed over the whole gene with a higher density in the β‐propeller and Sec39 domain as in the C‐terminal region (Figure S1). Thirteen variants have not been reported before (Table S1 provides genetic and clinical details on previously unpublished patients; Table S2 provides the genotype of all included individuals). Based on the type and localisation of the *NBAS* variants, 48 patients were assigned to the ILFS2 subgroup, 125 patients to the SOPH subgroup and 18 patients to the combined phenotype. Thirty‐nine patients could not be assigned to any of the three subgroups. Of these, 19 cases carried missense or in‐frame variants affecting two different protein segments. Ten patients presented variants affecting unclassified protein segments: namely, the N‐terminal segment (amino acid 1–85) and the segment between the β‐propeller and the sec39 domain (amino acid 447–721). In addition, ten patients had splice site variants or intronic variants.

### 3.3 Hepatic Phenotype

Of all 230 patients, the liver was the most frequently affected organ system (63.5%), followed by the nervous system (52.2%), skeleton (43.9%), immune system (37%), integument (33%) and endocrine system (7.8%). All patients in the ILFS2 and combined subgroups presented with hepatic abnormalities, while only a minority had liver abnormalities (33.6%) in the SOPH subgroup. Notably, the SOPH subgroup is dominated by the ethnic group of Yakuts sharing the same homozygous *NBAS* variant p.(Arg1914His), where liver abnormalities were reported to be a rare finding (11%).

In 41.3% of all patients, ALF criteria were met at least during a single episode of liver decompensation; however, the frequency of ALF differed between the subgroups: most patients in the combined (88.9%) and ILFS2 (91.7%) subgroups experienced at least one episode of ALF, and transaminases commonly normalised after the crises (combined: 14/17; ILFS2: 34/37). In contrast, 5.6% of patients in the SOPH group presented with ALF, and more than half of the SOPH patients with liver involvement (15/26) had continuously elevated transaminases. Furthermore, in the SOPH subgroup, chronic liver involvement is also reflected by permanent hepatomegaly (11/15 cases), whereas in the other subgroups, hepatomegaly is usually restricted to episodes of liver crises (combined subgroup: 9/14 cases; ILFS2 subgroup: 20/22 cases).

### 3.4 Characterisation of Liver Crises

The first liver crisis (ALF or elevation of liver transaminases (ELT) not meeting the ALF criteria) occurred at a median age of 0.9 years, with an age range between 2 days and 11 years. 88.8% of patients experienced their first liver crisis within the first 3 years of life, 97.2% before the age of five. Age at onset of liver crises differed between SOPH and ILFS subgroups (Figure S2A).

The most severe liver crisis occurred at a median age of 2.2 years, ranging from the 2 days to 12 years of age. 62.4% suffered their most severe liver crisis before the age of three, 88.2% before the age of five. However, there were no significant differences in age of most severe ELT/ALF between the subgroups (Figure S2B).

**FIGURE 1:**
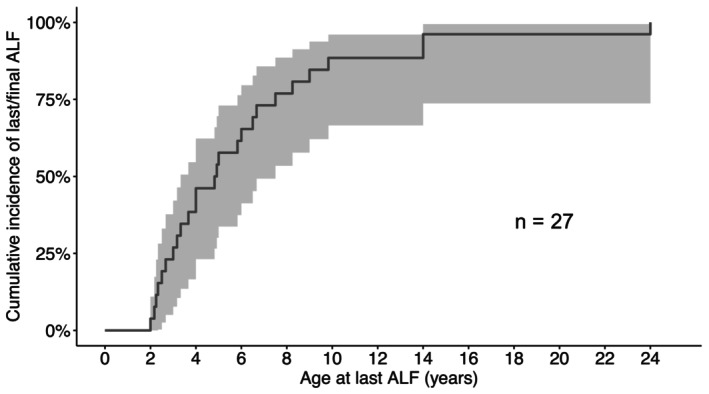
Cumulative incidence of patients who experienced ALF beeing without ALF for at least 5 years. Age relates to the last reported ALF. At the age of 10 years, 90% of the patients (*n* = 27) suffered their last ALF. ALF, acute liver failure.

In the analysis on age at last ALF, only patients who did not present with liver crises for 5 years or longer were included (*n* = 27). The median age at last ALF episode was 5 years. At the age of 10 years, 90% of the patients had suffered their last ALF (Figure 1). There was no significant difference between the subgroups in terms of age at last crisis (*p* = 0.73; log‐rank test; no data are available for SOPH patients without crises for at least 5 years). Regarding number of crises, an average of three episodes was reported (range 1–12 episodes, *n* = 83).

**FIGURE 2:**
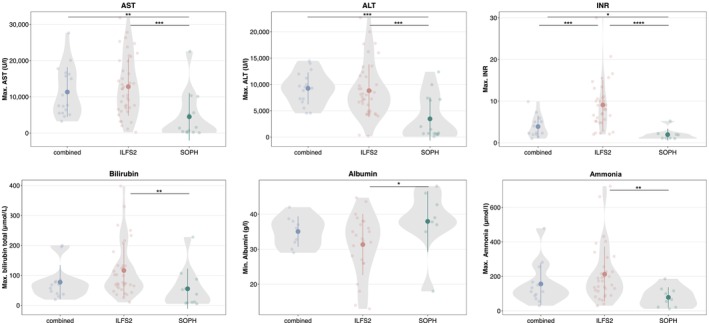
Maximum derangement of hepatic biomarkers by subgroup. Bold dots in the violin plots indicate the mean and bars the standard deviation. *p ≤ 0.05; **p ≤ 0.01; ***p ≤ 0.001; ****p ≤ 0.0001 Wilcoxon rank sum test. ALT, alanine transferase; AST, aspartate transferase; ILFS2, infantile liver failure syndrome type 2; INR, international normalised ratio; SOPH, short stature, optic atrophy and Pelger–Huët anomaly.

Patients in the ILFS2 subgroup had significantly higher levels of transaminase activities, bilirubin, INR, and ammonia as well as lower albumin concentrations compared to the SOPH subgroup (Figure 2). When comparing the SOPH and combined subgroups, it was found that only hepatic transaminases and INR were significantly higher in the combined subgroup. There was no significant difference in any of the laboratory parameters between the ILFS2 and combined subgroups.

Febrile infections were reported as a trigger of liver crises in all but one affected patient. In addition to febrile infections, reported triggers were postnatal catabolic period, fever post‐vaccination, chemotherapy, surgery, difficult intravenous sampling or catheterization, cold or hot temperatures, long‐distance travel, and the end of interferon therapy. All those triggers were reported in single episodes only (max. *n* = 2).

Recurrent vomiting during liver crisis was reported in 43.8% and HE in 45.2% of patients with liver involvement. HE also differed between subgroups: While only 2.8% of patients in the SOPH subgroup developed HE, this was observed in 89.7% of ILFS2 patients. The median grade of HE in the ILFS2 subgroup was III (*n* = 22).

### 3.5 Liver Biopsies

Histological data from liver biopsies were available for 64 patients (Table S3). The median age at liver biopsy was 1.9 years (range 0.08–14.83, *n* = 47). Fibrosis was found in 41.7% of all biopsies (*n* = 48) but without apparent difference between the three subgroups as well as in patients with (37.8%, *n* = 37) and without normalisation of transaminases (44.4%, *n* = 9) between crises. Age at biopsy did not have an effect on the fibrotic grade when comparing biopsies taken before and after 2 years of age. Steatosis was reported in 85% of cases (*n* = 52) and necrosis in 54% (*n* = 37) while disturbances of liver architecture and cirrhosis were not found. In one SOPH patient, cirrhosis was initially suspected because of increased liver stiffness in elastography, but this suspect was not confirmed by biopsy (NBAS 96, [44]).

### 3.6 Survival and Liver Transplantation

**FIGURE 3:**
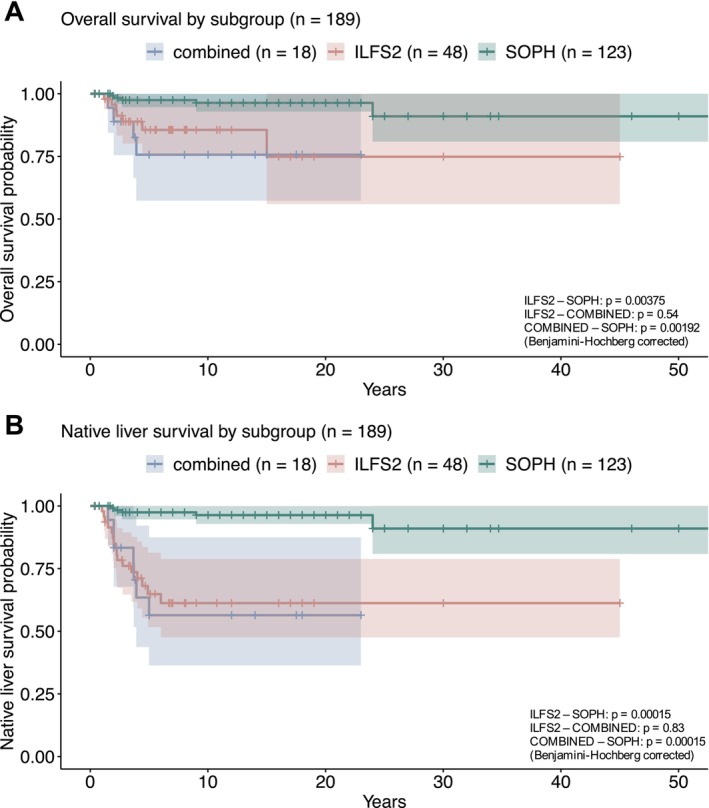
Survival and native liver survival by subgroups. ILFS2, infantile liver failure syndrome type 2; SOPH, short stature, optic atrophy and Pelger–Huët anomaly. *p* values by log rank test.

Twenty‐four (10.4%) of the observed patients with NBAS‐associated disease died during the study interval. The median age at death was 2.2 years (*n* = 24), ranging from 4 months to 24 years. The mortality rate was higher in the combined subgroup (22.2%) compared to the SOPH subgroup (4.8%; Figure 3A). Among those who died, ALF was the main cause of death (66.7%). Except for one patient who died during liver transplantation, ALF was the only cause of death in the combined and ILFS2 subgroups, while two of six patients in the SOPH subgroup died because of ALF and the remaining four because of epileptic state (*n* = 1), severe infection after liver transplantation (*n* = 1), and unknown cause of death (*n* = 2). One patient without subgroup assignment died of septic shock due to central line infection.

The rate of native liver survival (NLS) in the overall cohort was 83.9%. While the rate in the SOPH subgroup was 95.2%, only about two‐thirds of patients in the ILFS2 (66.7%) and combined (61.1%) subgroups survived with their native liver (chi‐square test: *p* < 0.0001; standardised Pearson residuals (> |1.96|)) (Figure 3B).

Liver transplants were performed in 16 patients (ILFS2: 10 patients, combined: 3 patients; SOPH: 1 patient, no subgroup: 2 patients). All patients were transplanted because of ALF; there were no transplants because of chronic liver dysfunction or cirrhosis. The median age at liver transplantation (first transplantation in patients with repeated liver transplantation; *n* = 2) was 2.15 years (range 0.08–5 years, *n* = 16). The median follow‐up after liver transplantation is 5 years (range 0–14 years, *n* = 16). The five‐year survival rate was 70% (*n* = 10). Five patients from the ILFS2 subgroup and two patients from the combined subgroup had no more bouts of liver dysfunction after transplantation, that is 54%.

Post‐transplant complications of varying severity occurred in nine patients: One patient from the ILFS2 subgroup and one patient without subgroup assignment showed intermittent episodes of slightly elevated transaminases in the context of febrile illnesses, but no recurrence of ALF. Another ILFS2 patient had normalised liver function but experienced repeated episodes of acute renal failure associated with an overdose of tacrolimus. Two ILFS2 patients had an increase in transaminases after transplantation, suspected to be a transplant rejection or drug‐induced liver injury. Liver function normalised, but one patient retained neurological impairments (intellectual disability, movement disorder, epilepsy), presumably not as a result of the liver transplant but of the severe liver failure he suffered at the time of transplantation. Two patients required a subsequent liver transplant. Two patients died shortly after the first transplant due to multi‐organ failure (no subgroup assignment) and severe infection (SOPH subgroup). These patients both had immunological abnormalities (hypogammaglobulinemia in both patients, reduced NK cells in the patient without subgroup assignment, NK cells not tested in the SOPH patient). Overall, however, immunological abnormalities were found with almost the same frequency in patients with an uneventful course (3/7) as in those with complications (4/9).

### 3.7 Prognostic Biomarkers

Next, we investigated whether subgroup assignment, laboratory parameters, and involvement of different organ systems were associated with significantly higher or lower rates of NLS and hence could serve as a prognostic biomarker (Table 2).

**TABLE 2:** Prognostic biomarkers in NBAS‐associated disease. Statistical significance was tested using the Mann–Whitney test for laboratory parameters and the chi‐square test for affected organ systems.

	NLS		*n* = 196	NNLS		*n* = 34	*p*
Subgroups							
Combined	61%			39%			
ILFS2	67%			33%			**< 0.0001**
SOPH	97%			3%			
	*Mean ± SD*	*Median*		*Mean ± SD*	*Median*		
Laboratory parameters							
Max. AST	9353 ± 944.5	7642	*n* = 74	12 125 ± 1386	11 500	*n* = 28	0.1178
Max. ALT	7027 ± 557.5	7174	*n* = 80	9141 ± 912.7	8815	*n* = 28	0.0543
Max. INR	4.84 ± 0.484	3.62	*n* = 58	10.25 ± 1.508	7.10	*n* = 20	**< 0.0001**
Max. total bilirubin	78.87 ± 8.661	67.0	*n* = 63	128.4 ± 20.44	86.5	*n* = 22	**0.0107**
Min. albumin	35.21 ± 1.051	36.7	*n* = 40	29.35 ± 2.288	31.0	*n* = 15	**0.0105**
Max. ammonia	154.3 ± 16.76	129	*n* = 55	256.9 ± 36.41	227	*n* = 21	**0.0047**
Max. PELD score	67.09 ± 10.44	15.1	*n* = 33	172.5 ± 26.50	38.6	*n* = 12	**< 0.0001**
Affected organ systems							
Liver	57%		*n* = 195	100%		*n* = 34	**< 0.0001**
Acute liver failure	34%		*n* = 191	91%		*n* = 33	**< 0.0001**
Nervous system	65%		*n* = 164	45%		*n* = 29	0.06
Skeletal system	72%		*n* = 123	45%		*n* = 29	**0.0086**
Immune system	60%		*n* = 122	43%		*n* = 28	0.1384
Low IgG	54%		*n* = 74	41%		*n* = 22	0.3355
NK cell deficiency	77%		*n* = 44	75%		*n* = 8	1
Integument	45%		*n* = 151	32%		*n* = 22	0.2612
Endocrine system	14%		*n* = 127	0%		*n* = 21	0.0767
Short stature	84%		*n* = 165	41%		*n* = 27	**< 0.0001**

*Note*: Bold was used for the *p* values when statistical significance was reached.

Abbreviations: ALT, alanine transferase; AST, aspartate transferase; IgG, immunoglobulin G; INR, international normalised ratio; NK cell natural killer cell; NLS, native liver survival; NNLS, non‐native liver survival; PELD, score paediatric end‐stage liver disease score.

Assignment to ILFS2 or combined subgroup is associated with a significantly lower rate of NLS compared to the SOPH subgroup (chi‐square test: *p* < 0.0001).

Regarding laboratory results, a statistically significant difference was found for maximum INR, total bilirubin, ammonia, PELD (paediatric end‐stage liver disease) score and minimum albumin. The mean value of the maximum AST and ALT was higher in the non‐native liver survival (NNLS) group, but not statistically significant.

The presence of ALF is significantly associated with a lower rate of NLS (*p* < 0.0001) (Figure S3).

Skeletal abnormalities and short stature are significantly associated with a better outcome regarding mortality, reflecting the better prognosis in SOPH patients with a multisystem phenotype and significantly fewer cases of liver failure. In the sub‐cohort of patients with ALF, there was no significant difference in organ involvement in the NLS and NNLS groups (data not shown).

### 3.8 Clinical Management

The use of a patient specific emergency protocol was reported in 63 patients. However, 75 patients received antipyretics. Anabolic infusions with glucose were used in 77 patients and lipids in 40 patients. The use of vitamin K is reported in 39 patients and fresh frozen plasma in 20 patients. N‐acetylcysteine (NAC) was used in 13 patients.

## 4 Discussion

Our study offers a comprehensive and detailed insight into the hepatic phenotype in NBAS‐associated disease, analysing data from 230 NBAS patients.

### 4.1 An Important and Global Cause of Paediatric ALF

Since the identification of NBAS‐associated disease as a cause of ALF in 2015 [5], the number of patients diagnosed with biallelic pathogenic *NBAS* variants has increased substantially. Two recent studies performing genetic analysis in paediatric patients with ALF of undetermined aetiology identified NBAS‐associated disease as the most frequent diagnosis [2, 3]. Together with the large number of published cases, this indicates that pathogenic *NBAS* variants are a relatively common cause of ALF in children that remain without a diagnosis before the introduction of next generation sequencing. NBAS‐associated disease occurs worldwide and has a relatively high genetic heterogeneity with 158 known pathogenic variants.

### 4.2 Hepatic Phenotype Ranging From Recurrent ALF to Chronic Liver Affection

NBAS‐associated disease can affect multiple organ systems, with the liver being the most frequently affected organ. Hepatic involvement is heterogeneous and differs significantly between the three subgroups proposed by Staufner et al. [7]: Recurrent ALF affects the majority of patients in the ILFS2 and combined subgroups. In the SOPH subgroup, liver involvement is reported in only one third of cases and in most patients, hepatic transaminases are constantly increased with a further increase during febrile infections, whereas liver function is usually not significantly impaired.

### 4.3 Genotype–Phenotype Association: Limitations and Open Questions

Although the phenotypic spectrum of the delineated subgroups differed, individual exceptions from the characteristic phenotypes can be found in all subgroups. A recent study showed that the genotype–phenotype correlation is strong in individuals with the same genotype [12], suggesting subsegments with different functions within the three protein domains. However, even in one family, siblings may present with different phenotypes (e.g., experiencing ALF or not). Incomplete penetrance, variable expressivity, genetic factors other than *NBAS* variants, epigenetic or environmental factors are possible explanations for the variable clinical presentation.

A major limitation of the genotype–phenotype association is that a significant proportion of patients (*n* = 39; 17%) cannot be unambiguously assigned to any subgroup. These patients present a rather inhomogeneous phenotype. Of particular interest is the previously unclassified and relatively large protein segment between the β‐propeller and the sec39 domain. All eight patients with at least one missense variant in this protein region presented with liver involvement and all but one developed ALF. However, with regard to extrahepatic symptoms, the phenotype is inconsistent, indicating that variants in this region of the protein have different functional consequences.

### 4.4 Liver Crises Occur Mainly in the First Years of Life, but Awareness Is Necessary at All Ages

Febrile infections are the main trigger of liver crisis and, as such, require immediate medical attention. Severe liver crises occur mainly in infancy. However, the large age range for ALF episodes (2 days–24 years) implies awareness of liver crises at all ages. Compared to other causes of ALF, NBAS patients show particularly high hepatic transaminases and INR levels [2, 74], which may guide the diagnostic work‐up to some extent.

Regarding the characteristics of the hepatic phenotype over time, our study is limited by the lack of complete data for each ELT/ALF episode (e.g., exact biochemical characterisation and age at each episode) and hence does not allow us to analyse the clinical course over time in relation to age. Further prospective studies are necessary to address this aspect and to enable separate analysis of ELT and ALF episodes. Nevertheless, with age of onset, the most severe episode, and the last ALF episode, we were able to define the vulnerable episode in the life of affected individuals.

### 4.5 A Single Case of Liver Cirrhosis

Continuously elevated transaminases reflect ongoing liver cell damage and raise the question of progressive liver disease. The prevalence of liver remodelling did not differ significantly between patients with and without normalisation of transaminases. Yet, the median age at liver biopsy in patients with continuously elevated transaminases was only 1.7 years, and only two patients with continuously elevated transaminases were older than 18 years: One SOPH patient is 19 years old; transaminases remain slightly elevated, but no liver function impairment or liver biopsy results were reported [33]. Particularly striking is the report of a 34‐year‐old SOPH patient who was diagnosed with liver fibrosis, portal hypertension, and oesophageal varices at the age of 20. At the age of 33, he developed a hepatic hydrothorax, and elastography showed hepatic fibrosis [44]. This single report illustrates the risk of chronic and progressive liver disease in NBAS patients. In general, the age of biopsy did not reflect a progressive liver remodelling, as there were no differences in fibrotic grades in biopsies taken in the first 2 years of life or after.

### 4.6 Liver Disease Is the Leading Cause of Death, but the Rate of Spontaneous Recovery Is Higher Than in Other Causes of ALF

The mortality rate of NBAS‐associated disease in this cohort is 10.4%, the rate of NNLS 16,1%. Although the involvement of other organ systems such as the skeletal, immune and nervous systems represents significant medical problems for affected NBAS patients, all but five fatal courses are attributed to liver affection. Focusing on NBAS patients with at least one episode of ALF, the mortality rate is 18.9% and the rate of NNLS is 32.6% (*n* = 95). Paediatric ALF studies including all causes of ALF report mortality rates between 30% and 47% and rates of NNLS between 48% and 76% [2, 74, 75, 76], while survival and need for liver transplantation vary widely depending on the underlying diagnosis. Hence, compared to other causes of ALF, NBAS‐associated disease has a promising rate of recovery.

### 4.7 Non‐Hepatic Disease Manifestation Is Not Associated With a Less Favourable Outcome

The hypothesis that affection of other organ systems, such as immunodeficiency, contributes to a more severe liver disease course seems reasonable. The fact that mortality is highest in the combined subgroup suffering from ALF and extrahepatic symptoms supports this hypothesis. However, no extrahepatic organ involvement was found to be a predictor of death or liver transplantation. Abnormality of the skeletal system and short stature are even associated with a higher rate of NLS. Yet a protective effect of skeletal and growth abnormalities seems unlikely. A more plausible explanation might be the high prevalence of these symptoms in the SOPH subgroup, with only a small percentage of patients suffering from ALF.

Abnormal excretion and synthesis parameters (ammonia, bilirubin, INR, albumin) are associated with a lower rate of NLS. The PELD score, calculated from the patients' albumin, bilirubin and INR, together with the age and degree of growth failure, has been developed to predict outcomes in children with chronic liver disease. In line with other studies using the PELD score in ALF [74, 77, 78], we found higher PELD scores in NBAS patients with poor outcomes.

### 4.8 Early Detection and Treatment of Liver Crises

Currently, there is no specific or curative therapy for NBAS‐associated disease. A recent study showed that knowledge of the genetic diagnosis and an emergency management protocol cannot prevent ALF, but there was a trend (without statistical significance) towards a milder course of liver crises [12]. Emergency management includes early antipyretic therapy, reported to reduce the severity of acute liver crises [6, 17, 34]. For antipyretic therapy, we recommend metamizole (8–16 mg/kg body weight as a single dose orally or parenterally at minimum intervals of 6 h, maximum single dose: 1000 mg), considering the increased risk of bleeding when using ibuprofen in the context of impaired liver function and the fact that paracetamol toxicity induces reactive oxidative species, which might play a role in the pathogenesis of the disease [4]. Other therapeutic strategies include the administration of anabolic infusions of glucose and lipids [6] and NAC against the production of reactive oxidative species [4]. Further studies are needed to evaluate the impact of therapeutic options, requiring detailed data on therapies and the course of individual crises, not assessed in this study.

### 4.9 No Recurrence of ALF After Liver Transplantation, but Risks and Consequences of Transplantation Must Be Considered

After transplantation, seven out of 16 patients showed normalisation of liver function and normal psychomotor development. Transplantation may even have an effect on other symptoms of NBAS‐associated disease: normalisation of growth after liver transplantation was reported in one patient. However, the underlying cause of short stature in NBAS patients is not yet understood. Nine transplanted patients suffered complications, but there was no recurrence of ALF. At 18.8%, the mortality rate in the group of transplant patients is almost twice as high as in the overall cohort. However, a particularly severe course can be assumed for patients who were transplanted. The 5‐year survival rate of 70% (*n* = 10) was low compared to liver transplantation in children in general [79]. Reasons remain to be elucidated and might be found in the multisystem involvement in NBAS‐associated disease. In summary, the decision regarding liver transplantation remains complex and should be made for each case individually.

### 4.10 Limitations Due to Variable Data Availability and Wide Age Range

There are several factors that limit the reliability of the data and results. Most importantly, data availability is relatively low for some parameters, for example, liver biopsy results. Furthermore, it can be assumed that laboratory results such as transaminases are more likely to be reported in publications if they are particularly high, and thus the calculated median may be overestimated. In addition, significant differences in age at last visit limit comparability, especially with regard to survival, the number of crises, or symptoms that develop with increasing age.

## 5 Conclusion

In conclusion, liver disease is the most prevalent organ affection in NBAS‐associated disease and by far the most important cause of death. This study highlights the variability and genotype–phenotype (subgroup) association of liver involvement, ranging from recurrent ALF in the ILFS2 and combined subgroups to chronic liver affection without ALF in SOPH patients. Differences within the subgroups and unclassified genotypes require further investigation. Liver crises are nearly always triggered by febrile infections and occur mainly during infancy; nevertheless, awareness of liver crises is important at all ages. NBAS‐related ALF has a higher rate of spontaneous recovery than other causes of ALF, although there is an important proportion of liver transplantation or death. With no reported recurrence of ALF, liver transplantation may be an option, especially in severe cases, although the 5‐year survival rate after liver transplant is relatively low at 70%. Current therapeutic strategies for liver crises focus on early antipyretic therapy, anabolic infusions with glucose and lipids, and the use of NAC. These recommendations are based on individual experiences and pathomechanistic considerations, with a need for systematic evaluation in the future.

## Author Contributions

D.L. and B.P. were responsible for the conception and the design of the study. Data acquisition was performed by B.P., D.L., N.H., C.A., S.M.B., F.D.B., E.C., C.D.‐V., A.D., D.F., N.H., R.H., M.H.J., M.L., El.L., Eb.L., H.N.Ö.M., A.P., B.P., P.S., J.E.S., L.L., T.S. and G.F.V. L.D.S., H.B., D.L. and S.F.G. were responsible for statistical analysis and figure composition. B.P. and D.L. drafted the article. S.K., G.F.H., H.P. and C.S. critically revised the content. All authors read and approved the final manuscript.

## Ethics Statement

All procedures followed were in accordance with the ethical standards of the responsible committee on human experimentation (institutional and national) and with the Helsinki Declaration of 1975, as revised in 2013. The study was approved by the ethical committee of the Medical Faculty Heidelberg (study number: S‐035_2014).

## Consent

Informed consent was obtained from all patients or their parents or caregivers in the case of minor patients, except for cases where patient data were retrieved from publications.

## Conflicts of Interest

The authors declare no conflicts of interest.

## Supporting information


**Figure S1.** Variant density and REVEL score across the NBAS protein. Schematic representation of the NBAS protein with the two known domains, the density of all variants found in this cohort and the REVEL score (prediction of the pathogenicity of missense variants across the protein based on a combination of scores).

**Figure S2.** Histogram of the age at first and most severe liver crisis classified by subgroups. (A) Age of onset differed between the subgroups (Kruskal–Wallis test: *p* = 0.0277). Mann Whitney test showed earlier age of onset in the SOPH subgroup compared with the ILFS2 subgroup (*p* = 0.0045). Other subgroup comparisons did not differ significantly. (B) Age at most severe ELT/ALF did not differ between the groups (Kruskal–Wallis test *p* = 0.2622). ALF, acute liver failure; ELT, elevated liver transaminases; ILFS2, infantile liver failure syndrome type 2; SOPH, short stature, optic atrophy and Pelger–Huët anomaly.

**Figure S3.** Kaplan–Meier plot of overall survival and native liver survival in patients with and without acute liver failure (ALF). Overall survival and rate of native liver survival differed significantly between the two groups (*p* < 0.0001).

**Table S1.** Genetic and clinical details on previously unpublished patients with NBAS‐associated disease (*n* = 13). ALF, acute liver failure; cELT continuously elevated liver transaminases; n.a., not applicable; ELT elevated liver transaminases.

**Table S2.** Genotype of the 230 included patients with NBAS associated disease. *IDs with a Y indicate patients from Yakutia.

**Table S3.** Age at liver biopsy and histological findings by subgroups. ILFS2, infantile liver failure syndrome type 2; SOPH, short stature, optic atrophy and Pelger–Huët anomaly.

**Appendix S1.** Supplemental methods.

## Data Availability

The data that support the findings of this study are available from the corresponding author upon reasonable request.

## References

[1] C. Della Corte , A. Mosca , A. Vania , A. Alterio , A. Alisi , and V. Nobili , “Pediatric Liver Diseases: Current Challenges and Future Perspectives,” Expert Review of Gastroenterology & Hepatology 10, no. 2 (2016): 255–265. 26641319 10.1586/17474124.2016.1129274

[2] D. Lenz , L. D. Schlieben , M. Shimura , et al., “Genetic Landscape of Pediatric Acute Liver Failure of Indeterminate Origin,” Hepatology 79, no. 5 (2024): 1075–1087. 37976411 10.1097/HEP.0000000000000684 PMC11020061

[3] R. Hegarty , P. Gibson , M. Sambrotta , et al., “Study of Acute Liver Failure in Children Using Next Generation Sequencing Technology,” Journal of Pediatrics 236 (2021): 124–130. 34023347 10.1016/j.jpeds.2021.05.041

[4] B. Peters , T. Dattner , L. D. Schlieben , T. Sun , C. Staufner , and D. Lenz , “Disorders of Vesicular Trafficking Presenting With Recurrent Acute Liver Failure: NBAS, RINT1, and SCYL1 Deficiency,” Journal of Inherited Metabolic Disease 48 (2024): e12707. 38279772 10.1002/jimd.12707 PMC11726157

[5] T. B. Haack , C. Staufner , M. G. Kopke , et al., “Biallelic Mutations in NBAS Cause Recurrent Acute Liver Failure With Onset in Infancy,” American Journal of Human Genetics 97, no. 1 (2015): 163–169. 26073778 10.1016/j.ajhg.2015.05.009 PMC4572578

[6] C. Staufner , T. B. Haack , M. G. Kopke , et al., “Recurrent Acute Liver Failure due to NBAS Deficiency: Phenotypic Spectrum, Disease Mechanisms, and Therapeutic Concepts,” Journal of Inherited Metabolic Disease 39, no. 1 (2016): 3–16. 26541327 10.1007/s10545-015-9896-7

[7] C. Staufner , B. Peters , M. Wagner , et al., “Defining Clinical Subgroups and Genotype‐Phenotype Correlations in NBAS‐Associated Disease Across 110 Patients,” Genetics in Medicine 22, no. 3 (2020): 610–621. 31761904 10.1038/s41436-019-0698-4

[8] N. Maksimova , K. Hara , I. Nikolaeva , et al., “Neuroblastoma Amplified Sequence Gene Is Associated With a Novel Short Stature Syndrome Characterised by Optic Nerve Atrophy and Pelger‐Huet Anomaly,” Journal of Medical Genetics 47, no. 8 (2010): 538–548. 20577004 10.1136/jmg.2009.074815 PMC2921285

[9] N. G. Segarra , D. Ballhausen , H. Crawford , et al., “NBAS Mutations Cause a Multisystem Disorder Involving Bone, Connective Tissue, Liver, Immune System, and Retina,” American Journal of Medical Genetics Part A 167A, no. 12 (2015): 2902–2912. 26286438 10.1002/ajmg.a.37338

[10] A. Khoreva , E. Pomerantseva , N. Belova , et al., “Complex Multisystem Phenotype With Immunodeficiency Associated With NBAS Mutations: Reports of Three Patients and Review of the Literature,” Frontiers in Pediatrics 8 (2020): 577. 33042920 10.3389/fped.2020.00577 PMC7522312

[11] S. Richards , N. Aziz , S. Bale , et al., “Standards and Guidelines for the Interpretation of Sequence Variants: A Joint Consensus Recommendation of the American College of Medical Genetics and Genomics and the Association for Molecular Pathology,” Genetics in Medicine 17, no. 5 (2015): 405–424. 25741868 10.1038/gim.2015.30 PMC4544753

[12] N. Hammann , D. Lenz , I. Baric , et al., “Impact of Genetic and Non‐Genetic Factors on Phenotypic Diversity in NBAS‐Associated Disease,” Molecular Genetics and Metabolism 141, no. 3 (2024): 108118. 38244286 10.1016/j.ymgme.2023.108118

[13] D. Lenz , J. Pahl , F. Hauck , et al., “NBAS Variants Are Associated With Quantitative and Qualitative NK and B Cell Deficiency,” Journal of Clinical Immunology 41, no. 8 (2021): 1781–1793. 34386911 10.1007/s10875-021-01110-7 PMC8604887

[14] R. H. Squires, Jr. , B. L. Shneider , J. Bucuvalas , et al., “Acute Liver Failure in Children: The First 348 Patients in the Pediatric Acute Liver Failure Study Group,” Journal of Pediatrics 148, no. 5 (2006): 652–658, 10.1016/j.jpeds.2005.12.051. 16737880 PMC2662127

[15] J. M. Capo‐Chichi , C. Mehawej , V. Delague , et al., “Neuroblastoma Amplified Sequence (NBAS) Mutation in Recurrent Acute Liver Failure: Confirmatory Report in a Sibship With Very Early Onset, Osteoporosis and Developmental Delay,” European Journal of Medical Genetics 58, no. 12 (2015): 637–641. 26578240 10.1016/j.ejmg.2015.11.005

[16] A. Megarbane , L. Samaras , R. Chedid , et al., “Developmental Delay, Dysmorphic Features, Neonatal Spontaneous Fractures, Wrinkled Skin, and Hepatic Failure: A New Metabolic Syndrome?,” American Journal of Medical Genetics Part A 146A, no. 24 (2008): 3198–3201. 19012336 10.1002/ajmg.a.32579

[17] M. Balasubramanian , J. Hurst , S. Brown , et al., “Compound Heterozygous Variants in NBAS as a Cause of Atypical Osteogenesis Imperfecta,” Bone 94 (2017): 65–74. 27789416 10.1016/j.bone.2016.10.023 PMC6067660

[18] F. Kortum , I. Marquardt , M. Alawi , et al., “Acute Liver Failure Meets SOPH Syndrome: A Case Report on an Intermediate Phenotype,” Pediatrics 139, no. 1 (2017): e20160550. 28031453 10.1542/peds.2016-0550

[19] F. S. Regateiro , S. Belkaya , N. Neves , et al., “Recurrent Elevated Liver Transaminases and Acute Liver Failure in Two Siblings With Novel Bi‐Allelic Mutations of NBAS,” European Journal of Medical Genetics 60 (2017): 426–432. 28576691 10.1016/j.ejmg.2017.05.005

[20] P. L. Calvo , F. Tandoi , T. B. Haak , et al., “NBAS Mutations Cause Acute Liver Failure: When Acetaminophen Is Not a Culprit,” Italian Journal of Pediatrics 43, no. 1 (2017): 88. 28946922 10.1186/s13052-017-0406-4 PMC5613325

[21] M. Y. Hasosah , A. I. Iskandarani , A. I. Shawli , A. F. Alsahafi , G. A. Sukkar , and M. A. Qurashi , “Neuroblastoma Amplified Sequence Gene Mutation: A Rare Cause of Recurrent Liver Failure in Children,” Saudi Journal of Gastroenterology 23, no. 3 (2017): 206–208. 28611345 10.4103/1319-3767.207714 PMC5470381

[22] V. Cardenas , F. DiPaola , S. D. Adams , A. M. Holtz , and A. Ahmad , “Acute Liver Failure Secondary to Neuroblastoma Amplified Sequence Deficiency,” Journal of Pediatrics 186 (2017): 179–182. 28410752 10.1016/j.jpeds.2017.03.040

[23] J. Q. Li , Y. L. Qiu , J. Y. Gong , et al., “Novel NBAS Mutations and Fever‐Related Recurrent Acute Liver Failure in Chinese Children: A Retrospective Study,” BMC Gastroenterology 17, no. 1 (2017): 77. 28629372 10.1186/s12876-017-0636-3 PMC5477288

[24] Z. D. Li , Y. C. Li , C. H. Shen , J. S. Wang , and X. B. Xie , “Liver Transplantation for the Treatment of Acute Liver Failure in 3 Cases With NBAS Gene Deficiency and Literature Review,” Zhonghua Er Ke Za Zhi = Chinese Journal of Pediatrics 61, no. 1 (2023): 66–69. 36594124 10.3760/cma.j.cn112140-20220627-00595

[25] A. Haskins‐Olney , ed., “NBAS‐Related SOPH Syndrome With Immunodeficiency in a North American Patient,” ACMG Annual Clinical Genetics Meeting, 2016.

[26] Y. Sunwoo , Y. M. Kim , E. N. Kim , S. H. Oh , and B. H. Lee , “Severe Form of Neuroblastoma Amplified Sequence Deficiency in an Infant With Recurrent Acute Liver Failure,” Pediatrics International 60, no. 3 (2018): 302–304. 29575310 10.1111/ped.13476

[27] Y. M. Kim , Y. J. Lee , J. H. Park , et al., “High Diagnostic Yield of Clinically Unidentifiable Syndromic Growth Disorders by Targeted Exome Sequencing,” Clinical Genetics 92, no. 6 (2017): 594–605. 28425089 10.1111/cge.13038

[28] J. Wang , Z. Pu , and Z. Lu , “Targeted Nextgeneration Sequencing Reveals Two Novel Mutations of NBAS in a Patient With Infantile Liver Failure syndrome2,” Molecular Medicine Reports 17, no. 2 (2018): 2245–2250. 29207168 10.3892/mmr.2017.8191 PMC5783466

[29] T. Y. He , N. Zhang , Y. Xia , Y. Luo , C. R. Li , and J. Yang , “Short Stature, Optic Nerve Atrophy and Pelger‐Huet Anomaly Syndrome With Antibody Immunodeficiency and Aplastic Anemia: A Case Report and Literature Review,” Zhonghua Er Ke Za Zhi = Chinese Journal of Pediatrics 55, no. 12 (2017): 942–946. 29262476 10.3760/cma.j.issn.0578-1310.2017.12.015

[30] E. Palagano , G. Zuccarini , P. Prontera , et al., “Mutations in the Neuroblastoma Amplified Sequence Gene in a Family Affected by Acrofrontofacionasal Dysostosis Type 1,” Bone 114 (2018): 125–136. 29929043 10.1016/j.bone.2018.06.013

[31] N. Mungan , D. Yildizdas , C. Arikan , et al., “Recurrent Acute Liver Failure in a Family With NBAS Gene Mutation and Successful Liver Transplantation: First Cases From Turkey,” Journal of Inborn Errors of Metabolism & Screening 5 (2017): 1–413.

[32] R. Rius , L. G. Riley , Y. Guo , et al., “Cryptic Intronic NBAS Variant Reveals the Genetic Basis of Recurrent Liver Failure in a Child,” Molecular Genetics and Metabolism 126, no. 1 (2019): 77–82. 30558828 10.1016/j.ymgme.2018.12.002

[33] B. Fischer‐Zirnsak , R. Koenig , F. Alisch , et al., “SOPH Syndrome in Three Affected Individuals Showing Similarities With Progeroid Cutis Laxa Conditions in Early Infancy,” Journal of Human Genetics 64 (2019): 609–616. 31015584 10.1038/s10038-019-0602-8

[34] S. Ono , J. Matsuda , E. Watanabe , et al., “Novel Neuroblastoma Amplified Sequence (NBAS) Mutations in a Japanese Boy With Fever‐Triggered Recurrent Acute Liver Failure,” Human Genome Variation 6 (2019): 2. 30622725 10.1038/s41439-018-0035-5 PMC6323122

[35] X. Li , Q. Cheng , N. Li , et al., “SOPH Syndrome With Growth Hormone Deficiency, Normal Bone Age, and Novel Compound Heterozygous Mutations in NBAS,” Fetal and Pediatric Pathology 37 (2018): 404–410. 30592236 10.1080/15513815.2018.1509406

[36] Q. Yao , W. Wu , and X. Luo , eds., SOPH Syndrome Causes Recurrent Acute Liver Failure With Onset in Infancy (ICIEM, 2017).

[37] D. Carli , E. Giorgio , F. Pantaleoni , et al., “NBAS Pathogenic Variants: Defining the Associated Clinical and Facial Phenotype and Genotype‐Phenotype Correlations,” Human Mutation 40 (2019): 721–728. 30825388 10.1002/humu.23734

[38] V. K. Konstantopoulou , D. M. Moeslinger , B. G. Goeschl , and A. Roscher , “Severe Hypoglycemia and Seizures as the First Signs in NBAS Deficiency,” Journal of Inherited Metabolic Disease 41, no. S1 (2018): S37–S219.

[39] F. Thuriot , C. Buote , E. Gravel , et al., “Clinical Validity of Phenotype‐Driven Analysis Software PhenoVar as a Diagnostic Aid for Clinical Geneticists in the Interpretation of Whole‐Exome Sequencing Data,” Genetics in Medicine 20, no. 9 (2018): 942–949. 29388948 10.1038/gim.2017.239

[40] S. Mallakmir , A. Nagral , A. Bagde , D. Mirza , R. Merchant , and V. Yewale , “Mutation in the Neuroblastoma Amplified Sequence Gene as a Cause of Recurrent Acute Liver Failure, Acute Kidney Injury, and Status Epilepticus,” Journal of Clinical and Experimental Hepatology 9 (2019): 753–756. 31889758 10.1016/j.jceh.2019.03.008 PMC6926219

[41] S. Ricci , L. Lodi , D. Serranti , et al., “Immunological Features of Neuroblastoma Amplified Sequence Deficiency: Report of the First Case Identified Through Newborn Screening for Primary Immunodeficiency and Review of the Literature,” Frontiers in Immunology 10 (2019): 1955. 31507590 10.3389/fimmu.2019.01955 PMC6718460

[42] J. Chavany , A. Cano , B. Roquelaure , et al., “Mutations in NBAS and SCYL1, Genetic Causes of Recurrent Liver Failure in Children: Three Case Reports and a Literature Review,” Archives de Pédiatrie 27, no. 3 (2020): 155–159. 32146038 10.1016/j.arcped.2020.01.003

[43] J. L. Gu , W. J. Wang , L. Li , Y. J. Zheng , and X. N. Mao , “A Novel Compound Heterozygous Mutation in NBAS Gene Causes SOPH Syndrome and Liver Function Damage,” Zhonghua Er Ke Za Zhi = Chinese Journal of Pediatrics 57, no. 6 (2019): 487–489. 31216810 10.3760/cma.j.issn.0578-1310.2019.06.018

[44] S. Suzuki , T. Kokumai , A. Furuya , et al., “A 34‐Year‐Old Japanese Patient Exhibiting NBAS Deficiency With a Novel Mutation and Extended Phenotypic Variation,” European Journal of Medical Genetics 63, no. 11 (2020): 104039. 32805445 10.1016/j.ejmg.2020.104039

[45] Z. D. Li , K. Abuduxikuer , J. Zhang , et al., “NBAS Disease: 14 New Patients, a Recurrent Mutation, and Genotype‐Phenotype Correlation Among 24 Chinese Patients,” Hepatology Research 50, no. 11 (2020): 1306–1315. 32812336 10.1111/hepr.13559

[46] W. Li , Y. Zhu , Q. Guo , and C. Wan , “Infantile Fever‐Triggered Acute Liver Failure Caused by Novel Neuroblastoma Amplified Sequence Mutations: A Case Report,” BMC Gastroenterology 20, no. 1 (2020): 308. 32957979 10.1186/s12876-020-01451-4 PMC7507814

[47] M. Ritelli , E. Palagano , V. Cinquina , et al., “Genome‐First Approach for the Characterization of a Complex Phenotype With Combined NBAS and CUL4B Deficiency,” Bone 140 (2020): 115571. 32768688 10.1016/j.bone.2020.115571

[48] J. Y. Thong , A. Halim , Z. Li , and M. Halim , “The Connection Between Neuroblastoma Amplified Sequence Gene (NBAS) and the Short Stature‐Optic‐Atrophy‐Pelger‐Huet Anomaly Syndrome (SOPH) Literature Review,” International Journal of Innovative Science and Research Technology 6, no. 2 (2021): 52–61.

[49] S. Krishnan , A. Rughani , A. Tsai , and S. Palle , “Novel Compound Heterozygous Variants in the NBAS Gene in a Child With Osteogenesis Imperfecta and Recurrent Acute Liver Failure,” BMJ Case Reports 14, no. 2 (2021): e234993, 10.1136/bcr-2020-234993. PMC7868262 33542026

[50] F. J. Cotrina‐Vinagre , M. E. Rodríguez‐García , E. Martín‐Hernández , et al., “Characterization of a Complex Phenotype (Fever‐Dependent Recurrent Acute Liver Failure and Osteogenesis Imperfecta) due to NBAS and P4HB Variants,” Molecular Genetics and Metabolism 133, no. 2 (2021): 201–210. 33707149 10.1016/j.ymgme.2021.02.007

[51] B. Jiang , F. Xiao , X. Li , Y. Xiao , Y. Wang , and T. Zhang , “Case Report: Pediatric Recurrent Acute Liver Failure Caused by Neuroblastoma Amplified Sequence (NBAS) Gene Mutations,” Frontiers in Pediatrics 8 (2020): 607005. 33520894 10.3389/fped.2020.607005 PMC7838493

[52] D. Geem , W. Jiang , H. B. Rytting , et al., “Resolution of Recurrent Pediatric Acute Liver Failure With Liver Transplantation in a Patient With NBAS Mutation,” Pediatric Transplantation 25, no. 7 (2021): e14084. 34288298 10.1111/petr.14084 PMC8515489

[53] Y. Lacassie , B. Johnson , G. Lay‐Son , et al., “Severe SOPH Syndrome due to a Novel NBAS Mutation in a 27‐Year‐Old Woman‐Review of This Pleiotropic, Autosomal Recessive Disorder: Mystery Solved After Two Decades,” American Journal of Medical Genetics Part A 182, no. 7 (2020): 1767–1775. 32297715 10.1002/ajmg.a.61597

[54] P. Lipiński , M. Greczan , D. Piekutowska‐Abramczuk , et al., “NBAS Deficiency due to Biallelic c.2809C > G Variant Presenting With Recurrent Acute Liver Failure With Severe Hyperammonemia, Acquired Microcephaly and Progressive Brain Atrophy,” Metabolic Brain Disease 36, no. 7 (2021): 2169–2172. 34427841 10.1007/s11011-021-00827-z PMC8437862

[55] F. Nazmi , E. Ozdogan , N. O. Mungan , and C. Arikan , “Liver Involvement in Neuroblastoma Amplified Sequence Gene Deficiency Is Not Limited to Acute Injury: Fibrosis Silently Continues,” Liver International 41, no. 10 (2021): 2433–2439. 34396667 10.1111/liv.15038

[56] Y. Cheng , Z. Xia , C. Huang , and H. Xu , “Case Report: A Novel Cause of Acute Liver Failure in Children: A Combination of Human Herpesvirus‐6 Infection and Homozygous Mutation in NBAS Gene,” Journal of Clinical Laboratory Analysis 36, no. 5 (2022): e24343. 35349761 10.1002/jcla.24343 PMC9102514

[57] A. B. Dirim , T. Kalayci , M. Guzel Dirim , et al., “A Mysterious Cause of Recurrent Acute Liver Dysfunction for Over a Decade,” Gastroenterology Report 10 (2022): goab053. 35382171 10.1093/gastro/goab053 PMC8973007

[58] L. S. Akesson , R. Rius , N. J. Brown , et al., “Distinct Diagnostic Trajectories in NBAS‐Associated Acute Liver Failure Highlights the Need for Timely Functional Studies,” JIMD Reports 63, no. 3 (2022): 240–249. 35433172 10.1002/jmd2.12280 PMC8995841

[59] T. Jiang , W. Ouyang , Y. Tan , L. Tang , H. Zhang , and S. Li , “Clinical Features and Genetic Testing of a Child With Hepatic Failure Syndrome Type 2,” Zhonghua Yi Xue Yi Chuan Xue Za Zhi = Zhonghua Yixue Yichuanxue Zazhi = Chinese Journal of Medical Genetics 39, no. 2 (2022): 181–184. 35076915 10.3760/cma.j.cn511374-20200718-00526

[60] J. Ji , M. Yang , J. Jia , et al., “A Novel Variant in NBAS Identified From an Infant With Fever‐Triggered Recurrent Acute Liver Failure Disrupts the Function of the Gene,” Human Genome Variation 10, no. 1 (2023): 13. 37055399 10.1038/s41439-023-00241-0 PMC10102179

[61] C. S. Priglinger , G. Rudolph , I. Schmid , et al., “Characterization of a Novel Non‐Canonical Splice Site Variant (c.886‐5T>A) in NBAS and Description of the Associated Phenotype,” Molecular Genetics & Genomic Medicine 11, no. 3 (2023): e2120. 36479642 10.1002/mgg3.2120 PMC10009903

[62] D. Petukhova , E. Gurinova , A. Sukhomyasova , and N. Maksimova , eds., “Identification of a Novel Compound Heterozygous Variant in NBAS Causing Bone Fragility by the Type of Osteogenesis Imperfecta,” International Symposium on Bioinformatics Research and Applications, Springer, 2020.

[63] H. Shabani‐Mirzaee , F. Sayarifard , A. Malekiantaghi , and K. Eftekhari , “Acute Infantile Liver Failure Syndrome Type 2 in a 2.5‐Year‐Old Boy: A Case Report,” Clinical Case Reports 11, no. 9 (2023): e7892. 37692149 10.1002/ccr3.7892 PMC10485241

[64] R. Brauner , J. Bignon‐Topalovic , A. Bashamboo , and K. McElreavey , “Pituitary Stalk Interruption Syndrome Is Characterized by Genetic Heterogeneity,” PLoS One 15, no. 12 (2020): e0242358. 33270637 10.1371/journal.pone.0242358 PMC7714207

[65] Y. Yang , X. Fei , F. Lei , L. Wang , X. Yu , and Y. Tang , “Autoimmune Hemolytic Anemia and Thrombocytopenia in a Chinese Patient With Heterozygous NBAS Mutations: Case Report,” Medicine 103, no. 12 (2024): e36975. 38517998 10.1097/MD.0000000000036975 PMC10956969

[66] A. Yadav , R. J. Hopkin , and E. K. Schorry , eds., “SOPH Syndrome: Multisystem Disorder With Facial Dysmorphism, Skeletal Dysplasia, Episodic Liver Failure, Immune Dysfunction and Intellectual Disability,” American Society of Human Genetics 2017 Meeting, Orlando, 2017.

[67] A. Rothenbuhler , J. Maluenda , C. Aumont , V. Picard , P. Bougneres , and J. Melki , eds., “NBAS Mutations, a New Monogenic Cause of DISOPHAL, a New Syndrome With Type 1 Diabetes (T1D),” in Hormone Research in Pædiatrics (Karger, 2016).

[68] J. Liu , Y. Zheng , J. Huang , et al., “Expanding the Genotypes and Phenotypes for 19 Rare Diseases by Exome Sequencing Performed in Pediatric Intensive Care Unit,” Human Mutation 42, no. 11 (2021): 1443–1460. 34298581 10.1002/humu.24266 PMC9292147

[69] G. F. Godinez‐Zamora , P. Baeza‐Capetillo , A. Villaseñor‐Dominguez , et al., eds., A Novel Mutation in NBAS Causes SOPH Syndrome (ASHG, 2018).

[70] R. R. Dayan , O. N. R. Bignall, II , S. Johnson , F. Flores , and O. Volovelsky , “Neuroblastoma Amplified Sequence Gene Mutations Inducing Acute Kidney and Liver Injury in an Adolescent Female,” Case Reports in Nephrology and Dialysis 10, no. 3 (2020): 117–123. 33173785 10.1159/000508784 PMC7588679

[71] Y. Seo , T. Y. Kim , D. Won , et al., “Genetic Spectrum and Characteristics of Autosomal Optic Neuropathy in Korean: Use of Next‐Generation Sequencing in Suspected Hereditary Optic Atrophy,” Frontiers in Neurology 13 (2022): 978532. 36071901 10.3389/fneur.2022.978532 PMC9441910

[72] C. Olimpio , I. Paramonov , L. Matalonga , et al., “Increased Diagnostic Yield by Reanalysis of Whole Exome Sequencing Data in Mitochondrial Disease,” Journal of Neuromuscular Diseases 11 (2024): 767–775. 38759022 10.3233/JND-240020 PMC11307028

[73] N. R. Maksimova , A. N. Nogovicina , K. A. Kurtanov , and E. I. Alekseeva , “Population Frequency and Age of Mutation G5741→A in Gene NBAS Which Is a Cause of SOPH Syndrome in Sakha (Yakutia) Republic,” Genetika 52, no. 10 (2016): 1194–1201. 29369590

[74] S. Kathemann , L. P. Bechmann , J. P. Sowa , et al., “Etiology, Outcome and Prognostic Factors of Childhood Acute Liver Failure in a German Single Center,” Annals of Hepatology 14, no. 5 (2015): 722–728. 26256901

[75] P. Durand , D. Debray , R. Mandel , et al., “Acute Liver Failure in Infancy: A 14‐Year Experience of a Pediatric Liver Transplantation Center,” Journal of Pediatrics 139, no. 6 (2001): 871–876. 11743517 10.1067/mpd.2001.119989

[76] R. T. Ng , K. S. Chew , C. L. Choong , et al., “Etiology, Outcome and Prognostic Indicators of Acute Liver Failure in Asian Children,” Hepatology International 16, no. 6 (2022): 1390–1397. 36131224 10.1007/s12072-022-10417-5

[77] M. C. Sanchez and D. E. D'Agostino , “Pediatric End‐Stage Liver Disease Score in Acute Liver Failure to Assess Poor Prognosis,” Journal of Pediatric Gastroenterology and Nutrition 54, no. 2 (2012): 193–196. 21886007 10.1097/MPG.0b013e3182349a04

[78] R. Núñez‐Ramos , S. Montoro , M. Bellusci , et al., “Acute Liver Failure: Outcome and Value of Pediatric End‐Stage Liver Disease Score in Pediatric Cases,” Pediatric Emergency Care 34, no. 6 (2018): 409–412. 29851917 10.1097/PEC.0000000000000884

[79] V. L. Ng , A. Fecteau , R. Shepherd , et al., “Outcomes of 5‐Year Survivors of Pediatric Liver Transplantation: Report on 461 Children From a North American Multicenter Registry,” Pediatrics 122, no. 6 (2008): e1128–e1135. 19047213 10.1542/peds.2008-1363

