# Supplementary figures and images for: Hepatic Phenotype in NBAS‐Associated Disease: Clinical Course, Prognostic Factors and Outcome in 230 Patients
Source: Liver Int. 2025 May 28;45(7):e70146. doi: 10.1111/liv.70146 (PMC12117995; doi:10.1111/liv.70146)

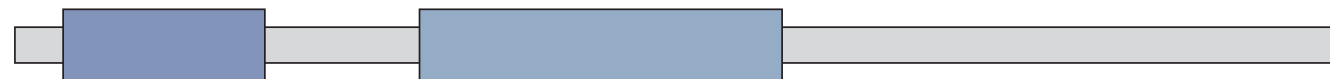

0 500 1000 1500 2000

Domain 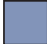 Beta-propeller domain 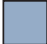 Sec39

Variant density

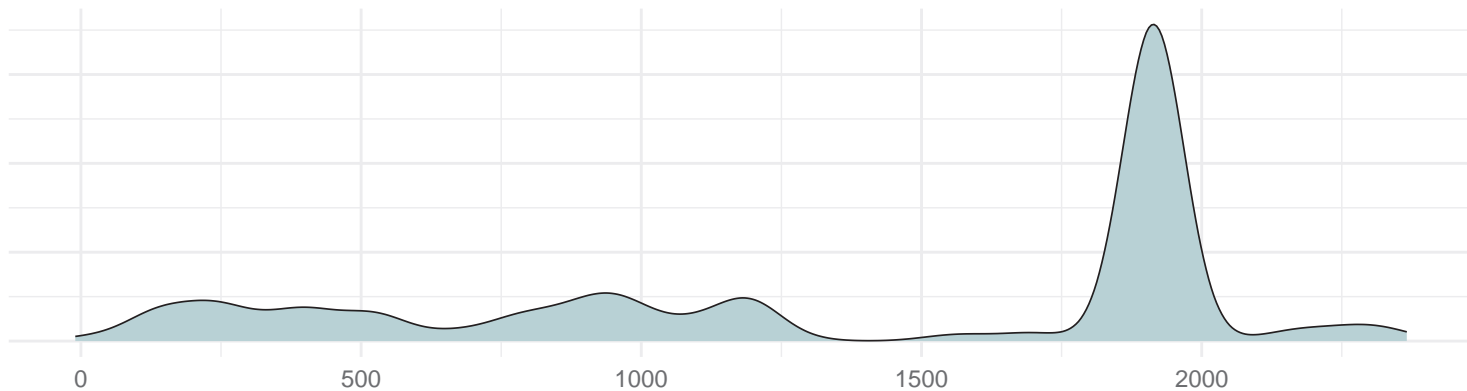

REVEL score

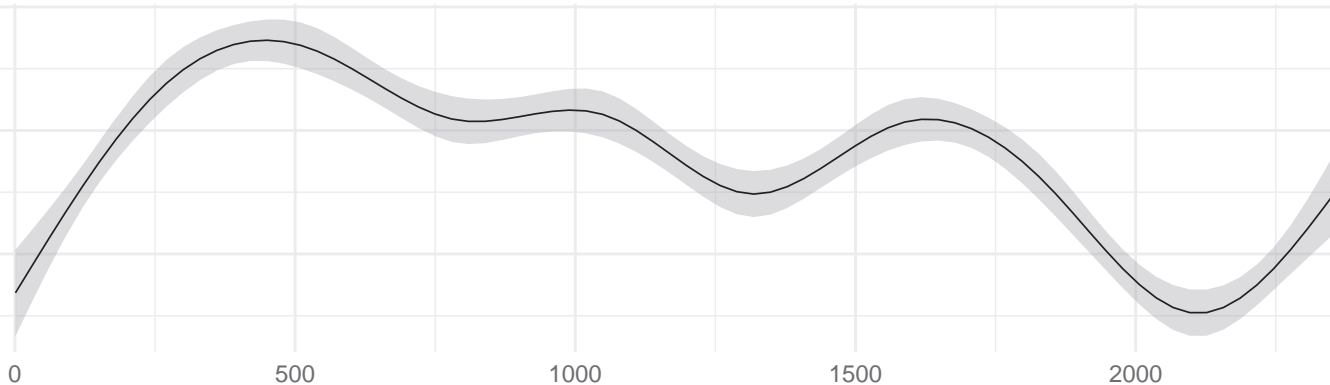

Protein Position

Supplement: Supplementary file 1 — Figure S1. Variant density and REVEL score across the NBAS protein. Schematic representation of the NBAS protein with the two known domains, the density of all variants found in this cohort and the REVEL score (prediction of the pathogenicity of missense variants across the protein based on a combination of scores). [file LIV-45-0-s002.pdf]

**A****Age of onset by subgroup (n = 77)**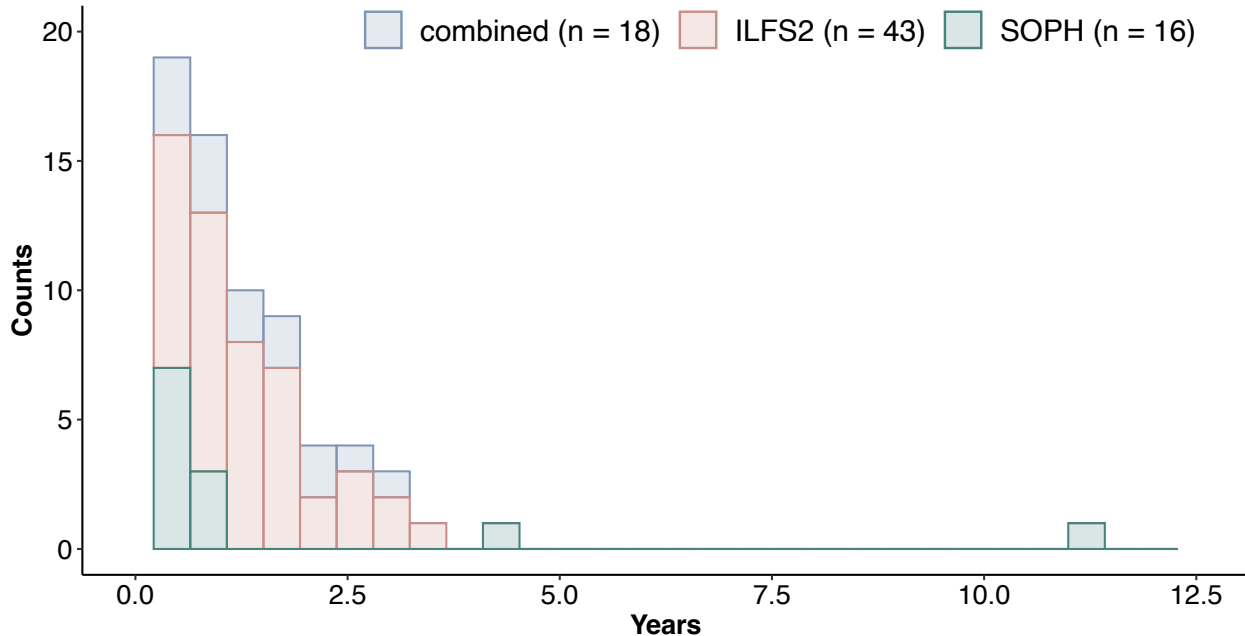**B****Age of most severe ELT/ALF (n = 70)**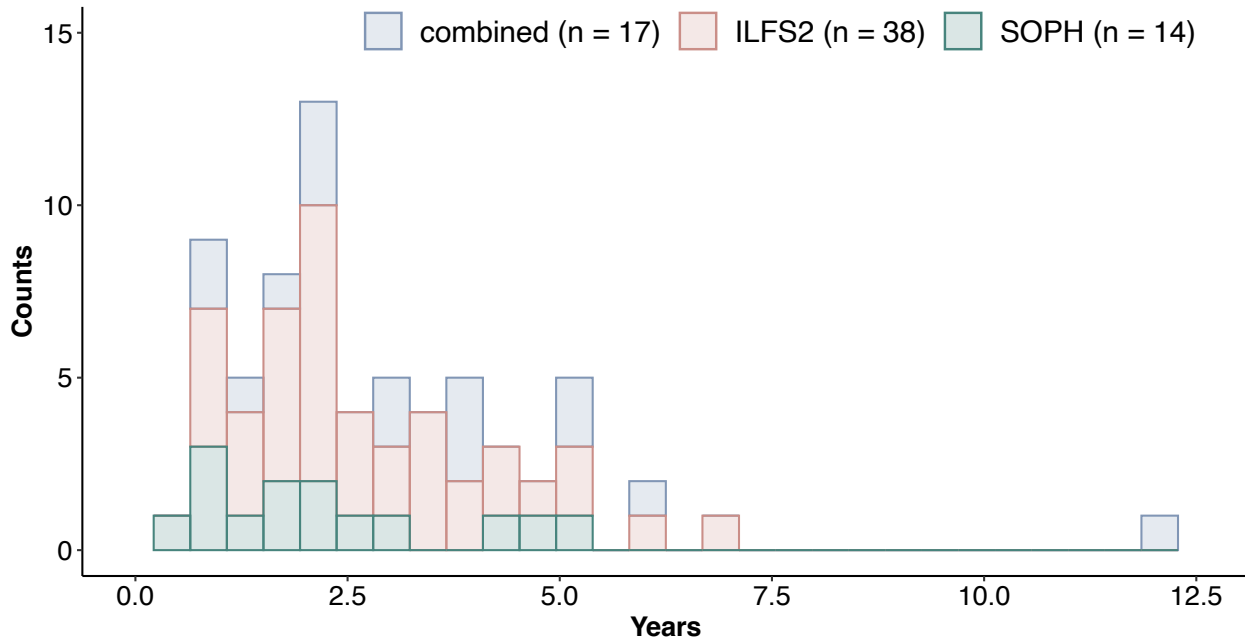

Supplement: Supplementary file 2 — Figure S2. Histogram of the age at first and most severe liver crisis classified by subgroups. (A) Age of onset differed between the subgroups (Kruskal–Wallis test: p = 0.0277). Mann Whitney test showed earlier age of onset in the SOPH subgroup compared with the ILFS2 subgroup (p = 0.0045). Other subgroup comparisons did not differ significantly. (B) Age at most severe ELT/ALF did not differ between the groups (Kruskal–Wallis test p = 0.2622). ALF, acute liver failure; ELT, elevated liver transaminases; ILFS2, infantile liver failure syndrome type 2; SOPH, short stature, optic atrophy and Pelger–Huët anomaly. [file LIV-45-0-s006.pdf]

# Native liver survival (n = 222)

ALF (n = 95) no-ALF (n = 127)

Native liver survival probability

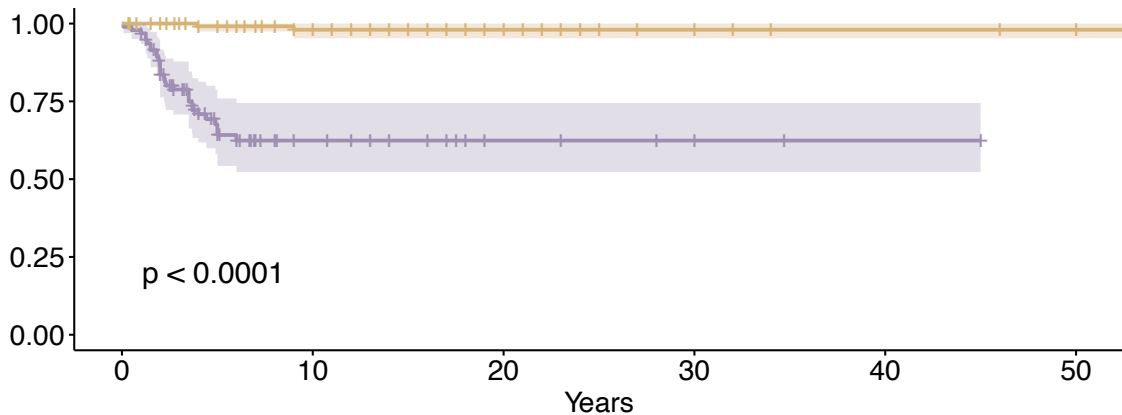

$p < 0.0001$

Number at risk

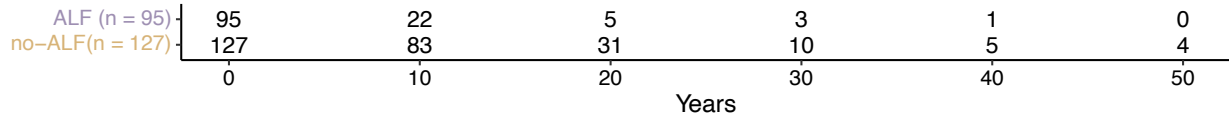

Supplement: Supplementary file 3 — Figure S3. Kaplan–Meier plot of overall survival and native liver survival in patients with and without acute liver failure (ALF). Overall survival and rate of native liver survival differed significantly between the two groups (p < 0.0001). [file LIV-45-0-s005.pdf]
