# Supplementary material for: Hepatic Phenotype in NBAS‐Associated Disease: Clinical Course, Prognostic Factors and Outcome in 230 Patients
Source: Liver Int. 2025 May 28;45(7):e70146. doi: 10.1111/liv.70146 (PMC12117995; doi:10.1111/liv.70146)
Supplement: Supplementary file 7 — Appendix S1. Supplemental methods. [file LIV-45-0-s003.docx]

**Supplemental Material**

**Supplemental methods:**

Exclusion criteria: Patient NBAS 61 has Williams-Beuren syndrome; NBAS 122 has cystic fibrosis (pathogenic homozygous *CFTR* variant), NBAS 123 carries two rare variants in the *KIAA0556* gene and a rare missense variant in *ROBO1*, both genes associated with clinical symptoms observed in this patient (64) and NBAS 161 carries an *ANKRD26* variant, associated with autosomal dominant thrombocytopenia observed in this patient [65]. One further patient (NBAS 154) was diagnosed with biliary atresia, underwent Kasai portoenterostomy and, later, required a liver transplant. As the hepatic presentation was primarily explained by the biliary atresia, the patient was excluded from analysis.

64. Brauner R, Bignon-Topalovic J, Bashamboo A, McElreavey K. Pituitary stalk interruption syndrome is characterized by genetic heterogeneity. PloS one. 2020;15(12):e0242358.

65. Yang Y, Fei X, Lei F, Wang L, Yu X, Tang Y. Autoimmune hemolytic anemia and thrombocytopenia in a Chinese patient with heterozygous NBAS mutations: Case report. Medicine (Baltimore). 2024;103(12):e36975.

**Supplemental figure legends:**

***Supplemental Figure S1. Variant density and REVEL score across the NBAS protein.*** *Schematic representation of the NBAS protein with the two known domains, the density of all variants found in this cohort and the REVEL score (prediction of the pathogenicity of missense variants across the protein based on a combination of scores).*

***Supplemental Figure S2.*** ***Histogram of the age at first and most severe liver crisis classified by subgroups.*** *(A) Age of onset differed between the subgroups (Kruskal Wallis test: p=0.0277). Mann Whitney test showed earlier age of onset in the SOPH subgroup compared with the ILFS2 subgroup (p=0.0045). Other subgroup comparisons did not differ significantly. (B) Age at most severe ELT/ALF did not differ between the groups (Kruskal-Wallis test p = 0.2622). ILFS2 infantile liver failure syndrome type 2; SOPH short stature, optic atrophy, and Pelger–Huët anomaly; ELT elevated liver transaminases; ALF acute liver failure.*

**Supplemental Figure S3. Kaplan-Meier plot of overall survival and native liver survival in patients with and without acute liver failure (ALF).** Overall survival and rate of native liver survival differed significantly between the two groups (p < 0.0001).

**Supplemental Table S1 Genetic and clinical details on previously unpublished patients with NBAS-associated disease** (n=13). n.a. not applicable; ALF acute liver failure; ELT elevated liver transaminases; cELT continuously elevated liver transaminases.

|  |  | **NBAS variants (NM_015909.3, NP_056993.2)** | | | | | | | **clinical phenotype** | | | | | | | |
| --- | --- | --- | --- | --- | --- | --- | --- | --- | --- | --- | --- | --- | --- | --- | --- | --- |
| **PID** | **sub-group** | **allele 1  (nucleotide change)** | **allele 1  (protein change)** | **region of NBAS affected by allele 1** | **allele 2  (nucleotid change)** | **allele 2  (protein change)** | **region of NBAS affected by allele 2** | **abnormality of the liver (HP:0001392)** | | **short stature (HP:0004322)** | **abnormality of the skeletal system (HP:0000924)** | **abnormality of the nervous system (HP:0000707)** | **abnormality of the integument (HP:0001574)** | **abnormality of the immune system (HP:0002715)** | **abnormality of the musculature (HP:0003011)** | **other abnormalities** |
| **NBAS 147** | **ß-propeller** | c.(451G>A) | p.(Glu151Lys) | ß-propeller | c.(680A>C) | p.(His227Pro) | ß-propeller | ALF | | no | no | no | no | no | no |  |
| **NBAS 160** |  | c.(809G>C) | p.(Gly270Ala) | ß-propeller | c.(1628_ 1629insA) | p.(Ser544fs*11) | n.a. | ALF | | no | no | no | no | reduced NK cells | no |  |
| **NBAS 149** |  | c.(1549C>T) | p.(Arg517Cys) | between ß-propeller and Sec39 | c.(4843C>T) | p.(Arg1615*) | n.a. | cELT | | yes | broad forehead, flat cheekbone, clinodactyly, delayed skeletal maturation, reduced bone mineral density, pathologic fracture, kyphosis, spinal stenosis | deficit of supratentorial white matter, motor delay | cutis laxa, reduced subcutaneous fat at trunk | decreased circulating IgG level, reduced NK cells | muscular hypotonia | pulmonary hypoplasia |
| **NBAS 154** |  | c.(2056C>T) | p.(Arg686Trp) | between ß-propeller and Sec39 | c.(3706C>T) | p.(Arg1236*) | n.a. | biliary atresia | | yes | reduced bone mineral density | no | no | decreased circulating IgG level, reduced NK cells | no | premature birth, Kasai portoenterostomy at 7 weeks, liver transplant at 6 months |
|  | **sec39** |  |  |  |  |  |  |  | |  |  |  |  |  |  |  |
| **NBAS 143** | **C-terminal** | c.(3860del) | p.(Leu1287*) | n.a. | c.(6859G>T) | p.(Asp2287Tyr) | C-terminal | ALF | | no | no | motor delay | no | reduced NK cells | no |  |
| **NBAS 151** |  | c.(6525C>G) | p.(His2175Gln) | C-terminal | c.(6787C>T) | p.(Arg2263*) | n.a. | ALF | | yes | triangular face, delayed closure of fontanel | motor delay | no | decreased circulating IgG level, reduced NK cells | muscular hypotonia | premature birth, failure to thrive |
| **NBAS 146** |  | c.(513+2T>C) | p.(?) | n.a. | c.(209+5G>T) | p.(?) | n.a. | ELT | | no | no | retinal dystrophy, nystagmus, seizures | no | reduced NK cells | no |  |
| **NBAS 148** |  | c.(6147-1G>T) | p.(?) | n.a. | c.(del exon 27-38) |  | n.a. | cELT | | yes | triangular face, delayed skeletal maturation, kyphosis | optic atrophy, deficit of supratentorial white matter, motor delay | reduced subcutaneous fat at trunk | decreased circulating IgG level, reduced NK cells | muscular hypotonia |  |
| **NBAS 155** |  | c.(2617C>T) | p.(Arg873Trp) | Sec39 | c.(5741G>A) | p.(Arg1914His) | C-terminal | ALF | | no | no | no | no | reduced NK cells | no |  |
| **NBAS 156** |  | c.(7003C>T) | p.(Arg2335Trp) | C-terminal | c.(1193A>G) | p.(Asp398Gly) | ß-propeller | ALF | | no | reduced bone mineral density | no | no | decreased circulating IgG level, reduced NK cells | no | died of multiple organ failure shortly after liver transplantation at the age of 2 months |
| **NBAS 157** |  | c.(3596G>A) | p.(Cys1199Tyr) | Sec39 | c.(3931+1_3932-1)_(4089+1_4090-1)del | p.(?) | n.a. | ALF | | no | no | learning disability | no | no | no | liver transplantation at the age of 1 year |
| **NBAS 158** |  | c.(3596G>A) | p.(Cys1199Tyr) | Sec39 | c.(3931+1_3932-1)_(4089+1_4090-1)del | p.(?) | n.a. | ALF | | no | no | learning disability | no | no | no | liver transplantation at the age of 6 years |
| **NBAS 159** |  | c.(3331delA) | p.(Thr1111 Hisfs*3) | n.a. | c.(6433_6572)_ (6573_6711) | p.(?) | n.a. | cELT, ALF | | no | no | no | no | reduced NK cells | no | ventricular septal defect, died at the age of 6 months of liver failure, septic shock and cardiac failure |

**Supplemental Table S2 Genotype of the 230 included patients with NBAS associated disease.***IDs with a Y indicate patients from Yakutia

| **NBAS ID*** | **Allel 1**  **(nucleotid change)** | **Allel 1**  **(protein change)** | **Allel 2**  **(nucleotid change)** | **Allel 2**  **(protein change)** | **Publication** |
| --- | --- | --- | --- | --- | --- |
| 1 | c.[558_560del] | p.[Ile187del] | c.[686dupT] | p.[Ser230Glnfs*4] | Haack et al., 2015 and Staufner et al., 2015 |
| 2 | c.[2708T>G] | p.[Leu903Arg] | c.[2708T>G] | p.[Leu903Arg] | Haack et al., 2015 and Staufner et al., 2015 |
| 3 | c.[603_605del] | p.[Leu202del] | c.[3164T>C] | p.[Leu1055Pro] | Haack et al., 2015 and Staufner et al., 2015 |
| 4 | c.[2708T>G] | p.[Leu903Arg] | c.[2827G>T] | p.[Glu943*] | Haack et al., 2015 and Staufner et al., 2015 |
| 5 | c.[3010C>T] | p.[Arg1004*] | c.[3164T>C] | p.[Leu1055Pro] | Haack et al., 2015 and Staufner et al., 2015 |
| 6 | c.[2827G>T] | p.[Glu943*] | c.[del exons 49-50 ] | p. [Val2145_Glu2237del] | Haack et al., 2015 and Staufner et al., 2015 |
| 7 | c.[1241C>T] | p. [Ser414Phe] | c.[2950delA] | p.[Ile984Leufs*8] | Staufner et al., 2020 |
| 8 | c.[1241C>T] | p.[Ser414Phe] | c.[del exon 48] | p.[del exon 48] | Staufner et al., 2020 |
| 9 | c.[2827G>T] | p.[Glu943*] | c.[2951T>G] | p.[Ile984Ser] | Staufner et al., 2020 |
| 10 | c.[1278A>C] | p.[Cys426Trp] | c.[del exons 39–40] | p.[Val1528Glyfs*2] | Haack et al., 2015 and Staufner et al., 2015 |
| 11 | c.[6840G>T] | p.[?] | c.[6840G>T] | p.[?] | Staufner et al., 2020 |
| 12 | c.[1550G>A] | p.[Arg517His] | c.[6805G>T] | p.[Glu2269*] | Staufner et al., 2020 |
| 13 | c.[1549C>T] | p.[Arg517Cys] | c.[5041_5048del] | p.[Ser1681Glnfs*37] | Staufner et al., 2020 |
| 14 | c.[686dupT] | p.[Ser230Glnfs*4] | c.[5761G>C] | p.[Ala1921Pro] | Staufner et al., 2020 |
| 15 | c.[1533_1545del] | p.[Ile512Thrfs*4] | c.[2951T>G] | p.[Ile984Ser] | Haack et al., 2015 and Staufner et al., 2015 |
| 16 | c.[1042C>T] | p.[Pro348Ser] | c.[2203-3C>G] | p.[?] | Haack et al., 2015 and Staufner et al., 2015 |
| 17 | c.[1042C>T] | p.[Pro348Ser] | c.[2203-3C>G] | p.[?] | Haack et al., 2015 and Staufner et al., 2015 |
| 18 | c.[1187G>A] | p.[Trp396*] | c.[2330C>A] | p.[Pro777His] | Haack et al., 2015 and Staufner et al., 2015 |
| 19 | c.[118-2A>G] | p.[?] | c.[2524G>T] | p.[Val842Phe] | Haack et al., 2015 and Staufner et al., 2015 |
| 20 | c.[686dup] | p.[Ser230Glnfs*4] | c.[3164T>C] | p.[Leu1055Pro] | Haack et al., 2015 and Staufner et al., 2015 |
| 21 | c.[173-2A>G] | p.[?] | c.[3363A>G] | p.[Ile1121Met] | Haack et al., 2015 and Staufner et al., 2015 |
| 22 | c.[284C>T] | p.[Ala95Val] | c.[850A>T] | p.[Lys284*] | Garcia Segarra et al., 2015 |
| 23 | c.[409C>T] | p.[Arg137Trp] | c.[1186T>A] | p.[Trp396Arg] | Garcia Segarra et al., 2015 |
| 24 | c.[409C>T] | p.[Arg137Trp] | c.[409C>T] | p.[Arg137Trp] | Mégarbané et al., 2008 and Capo-Chichi et al., 2015 |
| 25 | c.[409C>T] | p.[Arg137Trp] | c.[409C>T] | p.[Arg137Trp] | Mégarbané et al., 2008 and Capo-Chichi et al., 2015 |
| 26 | c.[409C>T] | p.[Arg137Trp] | c.[409C>T] | p.[Arg137Trp] | Mégarbané et al., 2008 and Capo-Chichi et al., 2015 |
| 27 | c.[3010C>T] | p.[Arg1004*] | c.[5741G>A] | p.[Arg1914His] | Balasubramanian et al., 2017 |
| 28 | c.[2032C>T] | p.[Gln678*] | c.[5741G>A] | p.[Arg1914His] | Balasubramanian et al., 2017 |
| 29 | c.[2827G>T] | p.[Glu943*] | c.[5741G>A] | p.[Arg1914His] | Kortüm et al., 2017 |
| 30 | c.[680A>C] | p.[His227Pro] | c.[1749G>A] | p.[Trp583*] | Regateiro et al., 2017 |
| 31 | c.[680A>C] | p.[His227Pro] | c.[1749G>A] | p.[Trp583*] | Regateiro et al., 2017 |
| 32 | c.[809G>C] | p.[Gly270Ala] | c.[2926del] | p.[Ser976Profs*16] | Calvo et al., 2017 |
| 33 | c.[2819A>C] | p.[His940Pro] | c.[2819A>C] | p.[His940Pro] | Hasosah et al., 2017 |
| 34 | c.[409C>T] | p.[Arg137Trp] | c.[758T>G] | p.[Val253Gly] | Cardenas et al., 2017 |
| 35 | c.[3596G>A] | p.[Cys1199Tyr] | c.[6611_6612insCA] | p.[Met2204Ilefs*3] | Li et al., 2017 |
| 36 | c.[586C>T] | p.[Gln196*] | c.[3596G>A] | p.[Cys1199Tyr] | Li et al., 2017 |
| 37 | c.[2407G>A] | p.[Glu803Lys] | c.[5389+1G>T] | p.[?] | Li et al., 2017; Zhongdie et al., 2022 |
| 40 | c.[336-3C>G] | p.[?] | c.[5123T>C] | p.[Leu1708Pro] | Haskins Olney et al., 2016 |
| 41 | c.[1339G>A] | p.[Glu447Lys] | c.[5454_5455delTT] | p.[Leu1818fs*16] | Sunwoo et al., 2017 |
| 42 | c.[5741G>A] | p.[Arg1914His] | c.[6572+1delG] | p.[?] | Kim et al., 2017 |
| 43 | c.[5741G>A] | p.[Arg1914His] | c.[6572+1delG] | p.[?] | Kim et al., 2017 |
| 44 | c.[209+1G>A] | p.[?] | c.[3596G>A] | p.[Cys1199Tyr] | Wang et al., 2018 |
| 45 | c.[5741C>T] | p[Arg1914His] | c.[6496-6497insA] | p.[Ser2166Phefs* 2] | He et al., 2017 |
| 46 | c.[284C>T] | p.[Ala95Val] | c.[2802G>A] | p.[Trp934*] | Staufner et al., 2020 |
| 47 | c.[6237-3C>G] | truncating mutation | c.[6237-3C>G] | truncating mutation | Palagano et al., 2018 (Prontera et al., 2011) |
| 48 | c.[6237-3C>G] | truncating mutation | c.[6237-3C>G] | truncating mutation | Palagano et al., 2018 |
| 50 | c.[1528C>T] | p.[Arg510*] | c.[5741G>A] | p.[Arg1914His] | Staufner et al., 2020 |
| 51 | c.[2191A>C] | p.[Thr731Pro] | c.[2191A>C] | p.[Thr731Pro] | Staufner et al., 2020 |
| 53 | c.[3602A>C] | p.[Gln1201Pro] | c.[3602A>C] | p.[Gln1201Pro] | Staufner et al., 2020 |
| 54 | c.[2819A>C] | p.[His940Pro] | c.[2819A>C] | p.[His940Pro] | Staufner et al., 2020 |
| 55 | c.[2330C>A] | p.[Pro777His] | c.[2330C>A] | p.[Pro777His] | Staufner et al., 2020 |
| 56 | c.[2330C>A] | p.[Pro777His] | c.[2330C>A] | p.[Pro777His] | Staufner et al., 2020 |
| 57 | c.[5547delC] | p.[Trp1850Glyfs*32] | c.[6966_6969delinsTC] | p.[Gln2322Hisfs*18] | Staufner et al., 2020 |
| 58 | c.[3386C>T] | p.[Ser1129Phe] | c.[3386C>T] | p.[Ser1129Phe] | Staufner et al., 2020 |
| 59 | c.[3386C>T] | p.[Ser1129Phe] | c.[3386C>T] | p.[Ser1129Phe] | Staufner et al., 2020 |
| 60 | c.[1342-6A>G] | p.[?] | c.[3534C>A] | p.[Ser1178Arg] | Staufner et al., 2020 |
| 62 | c.[5740C>G] | p.[Arg1914Gly] | c.[6877delC] | p.[Leu2293Cysfs*9] | Staufner et al., 2020 |
| 63 | c.[2423+404G>C] | p.[Arg809Phefs*10] | c.[2617C>T] | p.[Arg873Trp] | Rius et al., |
| 64 | c.[1550G>A] | p.[Arg517His] | c.[6805G>T] | p.[Glu2269*] | Staufner et al., 2020 |
| 65 | c.[405G>A] | p.[Trp135*] | c.[5741G>A] | p.[Arg1914His] | Fischer-Zirnsak et al., 2019 |
| 66 | c.[2950delA] | p.[Ile984Leufs*8] | c.[3158A>G] | p.[His1053Arg] | Fischer-Zirnsak et al., 2019 |
| 67 | c.[5741G>A] | p.[Arg1914His] | c.[6565_6566insT] | p.[Glu2189Valfs*7] | Fischer-Zirnsak et al., 2019 |
| 68 | c.[2809C>G] | p.[Pro937Ala] | c.[del exon 43-52] | p.[del exon 43-52] | Staufner et al., 2020 |
| 69 | c.[3164T>C] | p.[Leu1055Pro] | c.[del exon 42-44] | p.[del exon 42-44] | Staufner et al., 2020 |
| 70 | c.[1018G>C] | p.[Gly340Arg] | c.[2674G>T] | p.[Val892Phe] | Ono et al., 2019 |
| 77 | c.[500_501delTT] | p.[Phe167Cysfs*7] | c.[5752A > C] | p.[Thr1918Pro] | Li et al., 2018 |
| 78 | c.[5741C>T] | p.[Arg1914His] | c.[6496_6497insA] | p.[Ser2166Phefs*2] | Yao et al., 2017 |
| 79 | c.[1501C>T] | p.[Arg501*] | c.[6840G>A] | p.[?] | Carli et al., 2019 |
| 80 | c.[686dupT] | p.[Ser230fs*4] | c.[6840G>A] | p.[?] | Carli et al., 2019 |
| 82 | c.[812T>C] | p.[Leu271Pro] | c.[812T>C] | p.[Leu271Pro] | Staufner et al., 2020 |
| 83 | c.[2535G>T] | p.[Trp845Cys] | c.[5761G>C] | p.[Ala1921Pro] | Konstantopoulou et al., 2019 |
| 84 | c.[513+2T>C] | p.[?] | c.[3363A>G] | p.[Ile1121Met] | Staufner et al., 2020 |
| 85 | c.[686dupT] | p.[Ser230Glnfs*4] | c.[5741G>A] | p.[Arg1914His] | Thuriot et al., 2018; Buote, 2015 |
| 86 | c.[761G>A] | p.[Gly254Asp] | c.[761G>A] | p.[Gly254Asp] | Mallakmir et al., 2019 |
| 87 | c.[1948C>T] | p.[Pro650Ser] | c.[1948C>T] | p.[Pro650Ser] | Ricci et al., 2019 |
| 88 | c.[1342-6A>G] | p.[?] | c.[3534C>A] | p.[Ser1178Arg] | Lenz et al. 2021 |
| 89 | c.[3596G>A] | p.[Cys1199Tyr] | c.[209+1G>A] | p.[?] | Wang et al ,2018 |
| 90 | c.[6751_6754delCTCC] | p.[Leu2251Cysfs*5] | c.[3596-G>A] | p.[Cys1199Tyr] | Song et al., 2019 |
| 91 | c.[7041_7043delTCT] | p.[2346delLeu] | c.[3759delC] | p.[Thr1254Hisfs * 16] | Gu et al., 2019 |
| 94 | c.[2822G>A] | p.[Arg941His] | c.[2822G > A] | p.[Arg941His] | Chavany et al., 2020 |
| 95 | c.[2708T>G] | p.[Leu903Arg] | c.[2708T > G] | p.[Leu903Arg] | Chavany et al., 2020 |
| 96 | c.[5741G>A] | p.[Arg1914His] | c.[6433-2A>G] | p.[Ile2199_Asn2202delins16] p.[Ile2199Tyrfs*17] | Suzuki et al., 2020 |
| 97 | c.[426C>G] | p.[Tyr142*] | c.[3596G>A] | p.[Cys1199Tyr] | Die Li et al., 2020 |
| 98 | c.[501delT] | p.[Phe167Leufs*10] | c.[2570C>T] | p.[Ala857Val] | Die Li et al., 2020 |
| 99 | c.[648-1G>A] | p.[?] | c.[3596G>A] | p.[Cys1199Tyr] | Die Li et al., 2020 |
| 100 | c.[1018G>C] | p.[Gly340Arg] | c.[5740C>T] | p.[Arg1914Cys] | Die Li et al., 2020 |
| 101 | c.[2563_c.2577+5del] | p.[His855_Gln859del] | c.[3596G>A] | p.[Cys1199Tyr] | Die Li et al., 2020 |
| 102 | c.[2563_c.2577+5del] | p.[His855_Gln859del] | c.[3596G>A] | p.[Cys1199Tyr] | Die Li et al., 2020 |
| 103 | c.[3284G>A] | p.[Trp1095*] | c.[3596G>A] | p.[Cys1199Tyr] | Die Li et al., 2020 |
| 104 | c.[3432_3435dupCAGT] | p.[Ala1146Glnfs*14] | c.[6859G>T] | p.[Asp2287Tyr] | Die Li et al., 2020; Zhongdie et al., 2022 |
| 105 | c.[2822G>A] | p.[Arg941His] | c.[2423+404G>A] | p.[?] | Die Li et al., 2020 |
| 106 | c.[680_690dupACTGTTTCAGC] | p.[Phe231Thrfs*35] | c.[1361C>G] | p.[Pro454Arg] | Die Li et al., 2020 |
| 107 | c.[1028G>A] | p.[Ser343Asn] | c.[5741G>A] | p.[Arg1914His] | Die Li et al., 2020 |
| 108 | c.[1177_1182delinsAGATAGA] | p.[Val393Argfs*2] | c.[5752A>C] | p.[Thr1918Pro] | Die Li et al., 2020 |
| 109 | c.[2239 T>C] | p.[Tyr747Asp] | c.[7003C>T] | p.[Arg2335Trp] | Die Li et al., 2020 |
| 110 | c.[3115C>T] | p.[Gln1039*] | c.[7041_7043delTCT] | p.[2346delLeu] | Die Li et al., 2020 |
| 111 | c.[3386C>T] c.[1A>C] | p.[Ser1129Phe] p.[Met1Leu] | c.[875G>A] | [p.Gly292Glu] | Li et al., 2020 |
| 113 | c.[5741G>A] | p.[Arg1914His] | c.[del exon 35-47] | p.[del exon 35-47] | Khoreva et al., 2020 |
| 114 | c.[5741G>A] | p.[Arg1914His] | c.[del exon 35-47] | p.[del exon 35-47] | Khoreva et al., 2020 |
| 115 | c.[5741G>A] | p.[Arg1914His] | c.[1628_1629insA] | p.[Ser544fs] | Khoreva et al., 2020 |
| 116 | c.[686dup] | p.[Ser230Glnfs*4] | c.[2411A>G] | p.[Glu804Gly] | Ritelli et al., 2020 |
| 117 | c.[1549C>T] | p.[Arg517Cys] | c.[451G>A] | p.[Glu151Lys] | Krishnan et al., 2021 |
| 118 | c.[5139-T>G] | p.[?] | c.[2203-2A>G] | p.[?] | Thong et al., 2021 |
| 119 | c.[1265T>C] | p.[Leu422Pro] | c.[1549C>T] | p.[Arg517Cys] | Cotrina-Vinagre et al., 2021 |
| 120 | c.[5741 G>A] | p.[Arg1914His] | c.[686dupT] | p.[Ser230Glnfs*4] | Hammann et al., 2024 |
| 121 | c.[3596G>A] | p.[Cys1199Tyr] | ex.9del | p.[216-248 del] | Jiang et al., 2021 |
| 124 | c.[1549C>T] | p.[Arg517Cys] | c.[4646T>C] | p.[Leu1549Pro] | Geem et al., 2021 |
| 123 | c.[17C>A] | p.[Ser6*] | c.[5741G>A] | p.[Arg1914His] | Lacassie et al. 2020 |
| 129 | c.[2809C>G] | p.[Pro973Ala] | c.[2809C>G] | p.[Pro937Ala] | Lipinski et al., 2021 |
| 130 | c.[1556T>A] | p.[Val519Glu] | c.[1556T>A] | p.[Val519Glu] | Nazmi et al., 2021 |
| 131 | c.[307G>A] | p.[Ala103Thr] | c.[5119T>A] | p.[Phe1707Ile] | Nazmi et al., 2021 |
| 132 | c.[1628_1629InsA] | p.[Ser544Valfs*11] | c.[1226C>T] | p.[Ala409Val] | Nazmi et al., 2021 |
| 133 | c.[1187G>A] | p.[Trp396*] | c.[2330C>A] | p.[Pro777His] | Hammann et al., 2024 |
| 134 | c.[1187G>A] | p.[Trp396*] | c.[2330C>A] | p.[Pro777His] | Hammann et al., 2024 |
| 135 | c.[3602A>C] | p.[Gln1201Pro] | c.[3602A>C] | p.[Gln1201Pro] | Nazmi et al., 2021 |
| 136 | c.[3596G>A] | p.[Cys1199Tyr] | c.[3596G>A] | p.[Cys1199Tyr] | Cheng et al., 2022 |
| 137 | c.[1226C>T] | p.[Ala409Val] | c.[2479_2498del] | p.[?] | Dirim et al., 2021 |
| 138 | c.[2951T>G] | p.[Ile984Ser] | c.[406A>G] | p.[Arg136Gly] | Akesson et al. 2022 |
| 139 | c.[2951T>G] | p.[Ile984Ser] | c.[1213C>T] | p.[Arg405*] | Akesson et al. 2022 |
| 140 | c.[2951T>G] | p.[Ile984Ser] | c.[1213C>T] | p.[Arg405*] | Akesson et al. 2022 |
| 141 | c.[2746A>T] | p.[Arg916*] | c.[3596G>A] | p.[Cys1199Tyr] | Jiang et al., 2022 |
| 142 | c.[938_939delGC] | p.[Arg313Profs*2] | c.[1342T>C] | p.[Cys448Arg] | Ji et al., 2023 |
| 143 | c.[3860del] | p.[Leu1287*] | c.[6859G>T] | p.[Asp2287Tyr] | this publication |
| 144 | c.[5104del] | p.[Met1702*] | c.[886‐5T>A] | p.[?] | Priglinger et al., 2022 |
| 145 | c.[5104del] | p.[Met1702*] | c.[886‐5T>A] | p.[?] | Priglinger et al., 2022 |
| 146 | c.[513+2T>C] | p.[?] | c.[209+5G>T] | p.[?] | this publication |
| 147 | c.[451G>A] | p.[Glu151Lys] | c.[680A>C] | p.[His227Pro] | this publication |
| 148 | c.[6147-1G>T] | p.[?] | c.[del exon 27-38] | p.[del exon 27-38] | this publication |
| 149 | c.[1549C>T] | p.[Arg517Cys] | c.[4843C>T] | p.[Arg1615*] | this publication |
| 150 | c.[2407G>A] | p.[Glu803Lys] | c.[2401T>C] | p.[Trp801Gly] | Zhongdie et al., 2022 |
| 151 | c.[6525C>G] | p.[His2175Gln] | c.[6787C>T] | p.[Arg2263*] | this publication |
| 152 | c.[2535G>T] | p.[Trp845Cys] | c.[5741G>A] | p.[Arg1914His] | Petukhova et al., 2020 |
| 153 | c.[1725+1G>T] | p.[?] | c.[1725+1G>T] | p.[?] | Shabani-Mirzaee et al., 2023 |
| 155 | c.[2617C>T] | p.[Arg873Trp] | c.[5741G>A] | p.[Arg1914His] | this publication |
| 156 | c.[7003C>T] | p.[Arg2335Trp] | c.[1193A>G] | p.[Asp398Gly] | this publication |
| 157 | c.[3596G>A] | p.[Cys1199Tyr] | c.[3931+1_3932-1]_[4089+1_4090-1]del | p.[?] | this publication |
| 158 | c.[3596G>A] | p.[Cys1199Tyr] | c.[3931+1_3932-1]_[4089+1_4090-1]del | p.[?] | this publication |
| 159 | c.[3331delA] | p.[Thr1111Hisfs*3] | c.[6433_6572]_[6573_6711] | p.[?] | this publication |
| 160 | c.[809G>C] | p.[Gly270Ala] | c.[1628_1629insA] | p.[Ser544fs*11] | this publication |
| Y1 | c.[5741G>A] | p.[Arg1914His] | c.[5741G>A] | p.[Arg1914His] | Maksimova et al., 2010 |
| Y2 | c.[5741G>A] | p.[Arg1914His] | c.[5741G>A] | p.[Arg1914His] | Maksimova et al., 2010 |
| Y3 | c.[5741G>A] | p.[Arg1914His] | c.[5741G>A] | p.[Arg1914His] | Maksimova et al., 2010 |
| Y4 | c.[5741G>A] | p.[Arg1914His] | c.[5741G>A] | p.[Arg1914His] | Maksimova et al., 2010 |
| Y5 | c.[5741G>A] | p.[Arg1914His] | c.[5741G>A] | p.[Arg1914His] | Maksimova et al., 2010 |
| Y6 | c.[5741G>A] | p.[Arg1914His] | c.[5741G>A] | p.[Arg1914His] | Maksimova et al., 2010 |
| Y7 | c.[5741G>A] | p.[Arg1914His] | c.[5741G>A] | p.[Arg1914His] | Maksimova et al., 2010 |
| Y8 | c.[5741G>A] | p.[Arg1914His] | c.[5741G>A] | p.[Arg1914His] | Maksimova et al., 2010 |
| Y9 | c.[5741G>A] | p.[Arg1914His] | c.[5741G>A] | p.[Arg1914His] | Maksimova et al., 2010 |
| Y10 | c.[5741G>A] | p.[Arg1914His] | c.[5741G>A] | p.[Arg1914His] | Maksimova et al., 2010 |
| Y11 | c.[5741G>A] | p.[Arg1914His] | c.[5741G>A] | p.[Arg1914His] | Maksimova et al., 2010 |
| Y12 | c.[5741G>A] | p.[Arg1914His] | c.[5741G>A] | p.[Arg1914His] | Maksimova et al., 2010 |
| Y13 | c.[5741G>A] | p.[Arg1914His] | c.[5741G>A] | p.[Arg1914His] | Maksimova et al., 2010 |
| Y14 | c.[5741G>A] | p.[Arg1914His] | c.[5741G>A] | p.[Arg1914His] | Maksimova et al., 2010 |
| Y15 | c.[5741G>A] | p.[Arg1914His] | c.[5741G>A] | p.[Arg1914His] | Maksimova et al., 2010 |
| Y16 | c.[5741G>A] | p.[Arg1914His] | c.[5741G>A] | p.[Arg1914His] | Maksimova et al., 2010 |
| Y17 | c.[5741G>A] | p.[Arg1914His] | c.[5741G>A] | p.[Arg1914His] | Maksimova et al., 2010 |
| Y18 | c.[5741G>A] | p.[Arg1914His] | c.[5741G>A] | p.[Arg1914His] | Maksimova et al., 2010 |
| Y19 | c.[5741G>A] | p.[Arg1914His] | c.[5741G>A] | p.[Arg1914His] | Maksimova et al., 2010 |
| Y20 | c.[5741G>A] | p.[Arg1914His] | c.[5741G>A] | p.[Arg1914His] | Maksimova et al., 2010 |
| Y21 | c.[5741G>A] | p.[Arg1914His] | c.[5741G>A] | p.[Arg1914His] | Maksimova et al., 2010 |
| Y22 | c.[5741G>A] | p.[Arg1914His] | c.[5741G>A] | p.[Arg1914His] | Maksimova et al., 2010 |
| Y23 | c.[5741G>A] | p.[Arg1914His] | c.[5741G>A] | p.[Arg1914His] | Maksimova et al., 2010 |
| Y24 | c.[5741G>A] | p.[Arg1914His] | c.[5741G>A] | p.[Arg1914His] | Maksimova et al., 2010 |
| Y25 | c.[5741G>A] | p.[Arg1914His] | c.[5741G>A] | p.[Arg1914His] | Maksimova et al., 2010 |
| Y26 | c.[5741G>A] | p.[Arg1914His] | c.[5741G>A] | p.[Arg1914His] | Maksimova et al., 2010 |
| Y27 | c.[5741G>A] | p.[Arg1914His] | c.[5741G>A] | p.[Arg1914His] | Maksimova et al., 2010 |
| Y28 | c.[5741G>A] | p.[Arg1914His] | c.[5741G>A] | p.[Arg1914His] | Maksimova et al., 2010 |
| Y29 | c.[5741G>A] | p.[Arg1914His] | c.[5741G>A] | p.[Arg1914His] | Maksimova et al., 2010 |
| Y30 | c.[5741G>A] | p.[Arg1914His] | c.[5741G>A] | p.[Arg1914His] | Maksimova et al., 2010 |
| Y31 | c.[5741G>A] | p.[Arg1914His] | c.[5741G>A] | p.[Arg1914His] | Maksimova et al., 2010 |
| Y32 | c.[5741G>A] | p.[Arg1914His] | c.[5741G>A] | p.[Arg1914His] | Maksimova et al., 2010 |
| Y33 | c.[5741G>A] | p.[Arg1914His] | c.[5741G>A] | p.[Arg1914His] | Maksimova et al., 2010 |
| Y34 | c.[5741G>A] | p.[Arg1914His] | c.[5741G>A] | p.[Arg1914His] | Zhozhikov et al., 2023 |
| Y35 | c.[5741G>A] | p.[Arg1914His] | c.[5741G>A] | p.[Arg1914His] | Zhozhikov et al., 2023 |
| Y36 | c.[5741G>A] | p.[Arg1914His] | c.[5741G>A] | p.[Arg1914His] | Zhozhikov et al., 2023 |
| Y37 | c.[5741G>A] | p.[Arg1914His] | c.[5741G>A] | p.[Arg1914His] | Zhozhikov et al., 2023 |
| Y38 | c.[5741G>A] | p.[Arg1914His] | c.[5741G>A] | p.[Arg1914His] | Zhozhikov et al., 2023 |
| Y39 | c.[5741G>A] | p.[Arg1914His] | c.[5741G>A] | p.[Arg1914His] | Zhozhikov et al., 2023 |
| Y40 | c.[5741G>A] | p.[Arg1914His] | c.[5741G>A] | p.[Arg1914His] | Zhozhikov et al., 2023 |
| Y41 | c.[5741G>A] | p.[Arg1914His] | c.[5741G>A] | p.[Arg1914His] | Zhozhikov et al., 2023 |
| Y42 | c.[5741G>A] | p.[Arg1914His] | c.[5741G>A] | p.[Arg1914His] | Zhozhikov et al., 2023 |
| Y43 | c.[5741G>A] | p.[Arg1914His] | c.[5741G>A] | p.[Arg1914His] | Zhozhikov et al., 2023 |
| Y44 | c.[5741G>A] | p.[Arg1914His] | c.[5741G>A] | p.[Arg1914His] | Zhozhikov et al., 2023 |
| Y45 | c.[5741G>A] | p.[Arg1914His] | c.[5741G>A] | p.[Arg1914His] | Zhozhikov et al., 2023 |
| Y46 | c.[5741G>A] | p.[Arg1914His] | c.[5741G>A] | p.[Arg1914His] | Zhozhikov et al., 2023 |
| Y47 | c.[5741G>A] | p.[Arg1914His] | c.[5741G>A] | p.[Arg1914His] | Zhozhikov et al., 2023 |
| Y48 | c.[5741G>A] | p.[Arg1914His] | c.[5741G>A] | p.[Arg1914His] | Zhozhikov et al., 2023 |
| Y49 | c.[5741G>A] | p.[Arg1914His] | c.[5741G>A] | p.[Arg1914His] | Zhozhikov et al., 2023 |
| Y50 | c.[5741G>A] | p.[Arg1914His] | c.[5741G>A] | p.[Arg1914His] | Zhozhikov et al., 2023 |
| Y51 | c.[5741G>A] | p.[Arg1914His] | c.[5741G>A] | p.[Arg1914His] | Zhozhikov et al., 2023 |
| Y52 | c.[5741G>A] | p.[Arg1914His] | c.[5741G>A] | p.[Arg1914His] | Zhozhikov et al., 2023 |
| Y53 | c.[5741G>A] | p.[Arg1914His] | c.[5741G>A] | p.[Arg1914His] | Zhozhikov et al., 2023 |
| Y54 | c.[5741G>A] | p.[Arg1914His] | c.[5741G>A] | p.[Arg1914His] | Zhozhikov et al., 2023 |
| Y55 | c.[5741G>A] | p.[Arg1914His] | c.[5741G>A] | p.[Arg1914His] | Zhozhikov et al., 2023 |
| Y56 | c.[5741G>A] | p.[Arg1914His] | c.[5741G>A] | p.[Arg1914His] | Zhozhikov et al., 2023 |
| Y57 | c.[5741G>A] | p.[Arg1914His] | c.[5741G>A] | p.[Arg1914His] | Zhozhikov et al., 2023 |
| Y58 | c.[5741G>A] | p.[Arg1914His] | c.[5741G>A] | p.[Arg1914His] | Zhozhikov et al., 2023 |
| Y59 | c.[5741G>A] | p.[Arg1914His] | c.[5741G>A] | p.[Arg1914His] | Zhozhikov et al., 2023 |
| Y60 | c.[5741G>A] | p.[Arg1914His] | c.[5741G>A] | p.[Arg1914His] | Zhozhikov et al., 2023 |
| Y61 | c.[5741G>A] | p.[Arg1914His] | c.[5741G>A] | p.[Arg1914His] | Zhozhikov et al., 2023 |
| Y62 | c.[5741G>A] | p.[Arg1914His] | c.[5741G>A] | p.[Arg1914His] | Zhozhikov et al., 2023 |
| Y63 | c.[5741G>A] | p.[Arg1914His] | c.[5741G>A] | p.[Arg1914His] | Zhozhikov et al., 2023 |
| Y64 | c.[5741G>A] | p.[Arg1914His] | c.[5741G>A] | p.[Arg1914His] | Zhozhikov et al., 2023 |
| Y65 | c.[5741G>A] | p.[Arg1914His] | c.[5741G>A] | p.[Arg1914His] | Zhozhikov et al., 2023 |
| Y66 | c.[5741G>A] | p.[Arg1914His] | c.[5741G>A] | p.[Arg1914His] | Zhozhikov et al., 2023 |
| Y67 | c.[5741G>A] | p.[Arg1914His] | c.[5741G>A] | p.[Arg1914His] | Zhozhikov et al., 2023 |
| Y68 | c.[5741G>A] | p.[Arg1914His] | c.[5741G>A] | p.[Arg1914His] | Zhozhikov et al., 2023 |
| Y69 | c.[5741G>A] | p.[Arg1914His] | c.[5741G>A] | p.[Arg1914His] | Zhozhikov et al., 2023 |
| Y70 | c.[5741G>A] | p.[Arg1914His] | c.[5741G>A] | p.[Arg1914His] | Zhozhikov et al., 2023 |
| Y71 | c.[5741G>A] | p.[Arg1914His] | c.[5741G>A] | p.[Arg1914His] | Zhozhikov et al., 2023 |
| Y72 | c.[5741G>A] | p.[Arg1914His] | c.[5741G>A] | p.[Arg1914His] | Zhozhikov et al., 2023 |
| Y73 | c.[5741G>A] | p.[Arg1914His] | c.[5741G>A] | p.[Arg1914His] | Zhozhikov et al., 2023 |
| Y74 | c.[5741G>A] | p.[Arg1914His] | c.[5741G>A] | p.[Arg1914His] | Zhozhikov et al., 2023 |
| Y75 | c.[5741G>A] | p.[Arg1914His] | c.[5741G>A] | p.[Arg1914His] | Zhozhikov et al., 2023 |
| Y76 | c.[5741G>A] | p.[Arg1914His] | c.[5741G>A] | p.[Arg1914His] | Zhozhikov et al., 2023 |
| Y77 | c.[5741G>A] | p.[Arg1914His] | c.[5741G>A] | p.[Arg1914His] | Zhozhikov et al., 2023 |
| Y78 | c.[5741G>A] | p.[Arg1914His] | c.[5741G>A] | p.[Arg1914His] | Zhozhikov et al., 2023 |
| Y79 | c.[5741G>A] | p.[Arg1914His] | c.[5741G>A] | p.[Arg1914His] | Zhozhikov et al., 2023 |
| Y80 | c.[5741G>A] | p.[Arg1914His] | c.[5741G>A] | p.[Arg1914His] | Zhozhikov et al., 2023 |
| Y81 | c.[5741G>A] | p.[Arg1914His] | c.[5741G>A] | p.[Arg1914His] | Zhozhikov et al., 2023 |
| Y82 | c.[5741G>A] | p.[Arg1914His] | c.[5741G>A] | p.[Arg1914His] | Zhozhikov et al., 2023 |
| Y83 | c.[5741G>A] | p.[Arg1914His] | c.[5741G>A] | p.[Arg1914His] | Zhozhikov et al., 2023 |
| Y84 | c.[5741G>A] | p.[Arg1914His] | c.[5741G>A] | p.[Arg1914His] | Zhozhikov et al., 2023 |
| Y85 | c.[5741G>A] | p.[Arg1914His] | c.[5741G>A] | p.[Arg1914His] | Zhozhikov et al., 2023 |
| Y86 | c.[5741G>A] | p.[Arg1914His] | c.[5741G>A] | p.[Arg1914His] | Zhozhikov et al., 2023 |
| Y87 | c.[5741G>A] | p.[Arg1914His] | c.[5741G>A] | p.[Arg1914His] | Zhozhikov et al., 2023 |
| Y88 | c.[5741G>A] | p.[Arg1914His] | c.[5741G>A] | p.[Arg1914His] | Zhozhikov et al., 2023 |
| Y89 | c.[5741G>A] | p.[Arg1914His] | c.[5741G>A] | p.[Arg1914His] | Zhozhikov et al., 2023 |
| Y90 | c.[5741G>A] | p.[Arg1914His] | c.[5741G>A] | p.[Arg1914His] | Zhozhikov et al., 2023 |
| Y91 | c.[5741G>A] | p.[Arg1914His] | c.[5741G>A] | p.[Arg1914His] | Zhozhikov et al., 2023 |

**Supplemental Table S3 Age at liver biopsy and histological findings by subgroups.**
ILFS2 infantile liver failure syndrome type 2, SOPH short stature, optic atrophy, and Pelger–Huët anomaly.

| **Groups** | **Number of patients** | **Age at liver biopsy in years** median (range) | |  |  |  |  |  |  |  |
| --- | --- | --- | --- | --- | --- | --- | --- | --- | --- | --- |
| all | 64 | 2 (0.08 - 14.83) | | n = 47 |  |  |  |  |  |  |
| no subgroup | 14 | 1.8 (0.08 - 6.92) | | n = 12 |  |  |  |  |  |  |
| combined | 14 | 2.2 (0.67 - 14.83) | | n = 12 |  |  |  |  |  |  |
| ILFS2 | 29 | 1.7 (0.5 - 6) |  | n = 17 |  |  |  |  |  |  |
| SOPH | 7 | 1.1 (0.42 - 5.08) | | n = 6 |  |  |  |  |  |  |
| **Fibrosis** |  | No fibrosis |  | Portal fibrosis |  | Periportal fibrosis/ formation of septa | | Septa forming fibrosis with architecture disturbances | | Cirrhosis |
| all | 48 | 28 | 58% | 10 | 21% | 10 | 21% |  | 0 | 0 |
| no subgroup | 10 | 2 | 20% | 4 | 40% | 4 | 40% |  | 0 | 0 |
| combined | 9 | 7 | 78% | 2 | 22% | 0 | 0% |  | 0 | 0 |
| ILFS2 | 23 | 14 | 61% | 3 | 13% | 6 | 26% |  | 0 | 0 |
| SOPH | 6 | 5 | 83% | 1 | 17% | 0 | 0% |  | 0 | 0 |
| **Steatosis** |  | No steatosis |  | Microvesicular steatosis | | Macrovesicular steatosis | | Micro- and macrovesicular steatosis | |  |
| all | 52 | 8 | 15% | 29 | 56% | 7 | 13% | 8 | 15% |  |
| no subgroup | 13 | 0 | 0% | 6 | 46% | 4 | 31% | 3 | 23% |  |
| combined | 10 | 1 | 10% | 6 | 60% | 2 | 20% | 1 | 10% |  |
| ILFS2 | 23 | 6 | 26% | 14 | 61% | 0 | 0% | 3 | 13% |  |
| SOPH | 6 | 1 | 17% | 3 | 50% | 1 | 17% | 1 | 17% |  |
| **Necrosis** |  | No cell death |  | Single cell necrosis | | Group cell necrosis | |  |  |  |
| all | 37 | 17 | 46% | 10 | 27% | 10 | 27% |  |  |  |
| no subgroup | 8 | 4 | 50% | 3 | 38% | 1 | 13% |  |  |  |
| combined | 10 | 4 | 40% | 3 | 30% | 3 | 30% |  |  |  |
| ILFS2 | 15 | 7 | 47% | 2 | 13% | 6 | 40% |  |  |  |
| SOPH | 4 | 2 | 50% | 2 | 50% | 0 | 0% |  |  |  |
